# Supplementary material for: Influence of geographic origin and tissue type on the medicinal chemical compounds of Semiliquidambar cathayensis
Source: PeerJ. 2023 Jun 6;11:e15484. doi: 10.7717/peerj.15484 (PMC10252815; doi:10.7717/peerj.15484)
Supplement: Supplemental Information 1 [file peerj-11-15484-s001.docx]

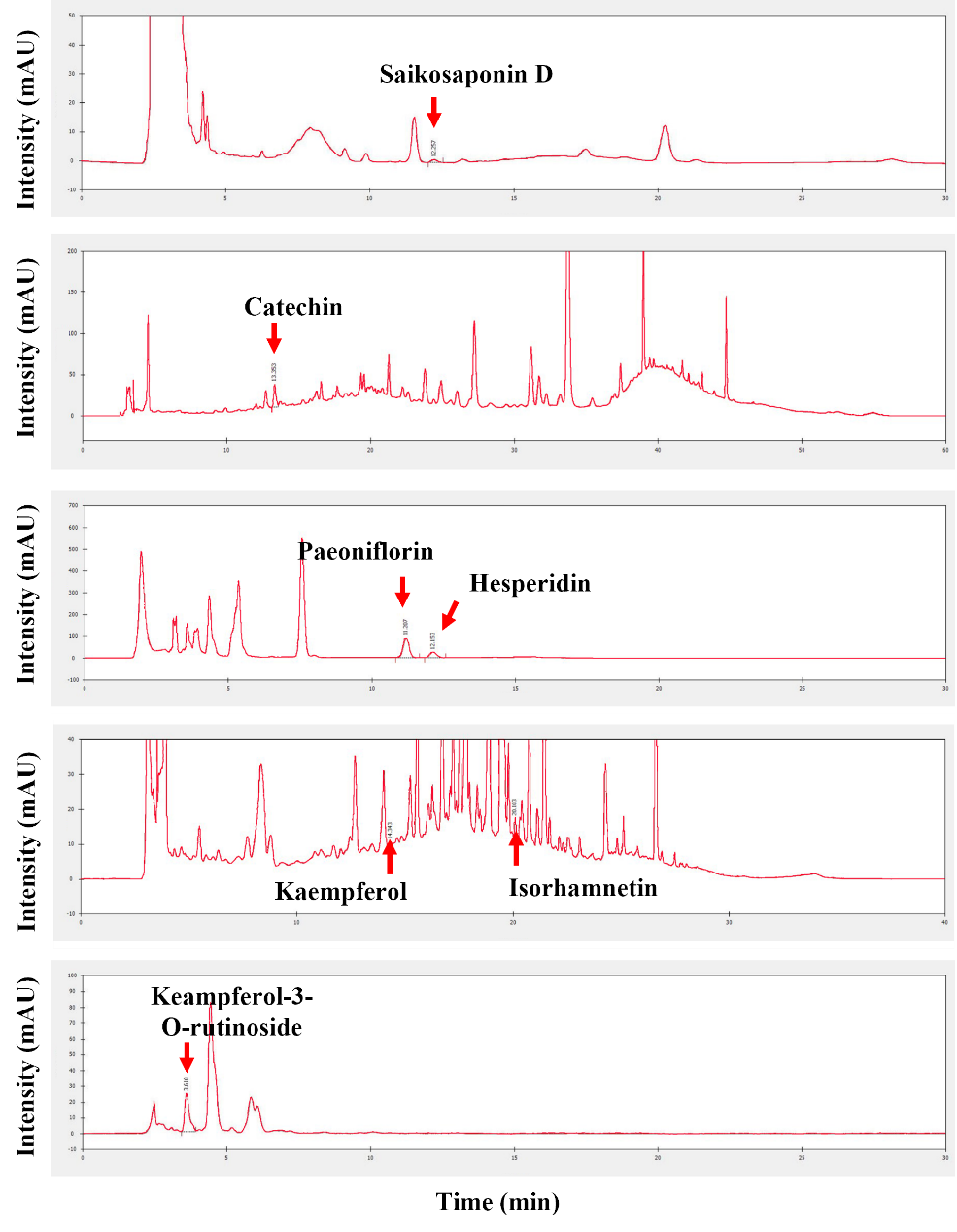


**Fig 1 Separation of chromatograms of various compounds in samples by HPLC method.**

- TIC MRM (** -> **) STD8.d (STD8) x103

7.5

7

6.5

6

5.5

5

4.5

4

3.5

3

2.5

2

1.5

1

2

3

4

5

6

7

8

9

10

11

12

13

14 15

16

Acquisition Time (min)

# Ursolic acid

# Oleanolic acid

Counts

**Figure 2 Sample Chromatogram (for the detection of ursolic acid and oleanolic acid)**

**c**

- MRM (455.3 -> 455.3) STD8.d x102

5

4.8

4.6

4.4

4.2

4

3.8

3.6

3.4

3.2

3

2.8

2.6

2.4

2.2

2

1.8

1.6

1.4

1.2

1

0.8

0.6

0.4

13.611 min.

11.8 12 12.2 12.4 12.612.8 13 13.2 13.4 13.6 13.8 14 14.2 14.4 14.6 14.8 15 15.215.4

Acquisition Time (min)

455.3 -> 455.3, 455.3 -> 407.3

x102 Ratio = 1.7 (163.1 %)

2.6

2.4

2.2

2

1.8

1.6

1.4

1.2

1

0.8

0.6

0.4

0.2

0

-0.2

11.8 12 12.2 12.4 12.6 12.8 13 13.2 13.4 13.6 13.8 14 14.214.414.614.8 15 15.2 15.4

Acquisition Time (min)

Counts

Relative Abundance (%)

Counts


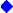


- MRM (13.478-13.688 min, 19 scans) (455.3 -> **) STD8.d x103

5.2

5

4.8

4.6

4.4

4.2

4

3.8

3.6

3.4

3.2

3

2.8

2.6

2.4

2.2

2

1.8

1.6

1.4

1.2

1

0.8

0.6

0.4

0.2

0

455.3

407.3

405 410 415 420 425 430 435 440 445 450 455

Mass-to-Charge (m/z)

**b**

**a**

**Figure 3 Multiple-reaction monitoring (a,b) and mass spectrum (c) of ursolic acid in the sample**

**c**

Relative Abundance (%)

Counts


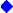


- MRM (13.688-13.924 min, 22 scans) (455.3 -> **) STD8.d x103

5.6

5.4

5.2

5

4.8

4.6

4.4

4.2

4

3.8

3.6

3.4

3.2

3

2.8

2.6

2.4

2.2

2

1.8

1.6

1.4

1.2

1

0.8

0.6

0.4

0.2

0

455.3

407.3

405 410 415 420 425 430 435 440 445 450 455

Mass-to-Charge (m/z)

455.3 -> 455.3, 455.3 -> 407.3

x102 Ratio = 0.3 (24.1 %) 2.5

2.4

2.3

2.2

2.1

2

1.9

1.8

1.7

1.6

1.5

1.4

1.3

1.2

1.1

1

0.9

0.8

0.7

0.6

0.5

0.4

0.3

0.2

0.1

0

-0.1

-0.2

-0.3

12 13 14 15

Acquisition Time (min)

- MRM (455.3 -> 455.3) STD8.d x102

5

4.8

4.6

4.4

4.2

4

3.8

3.6

3.4

3.2

3

2.8

2.6

2.4

2.2

2

1.8

1.6

1.4

1.2

1

0.8

0.6

0.4

13.611 min.

11.8 12 12.2 12.4 12.612.8 13 13.2 13.4 13.6 13.8 14 14.2 14.4 14.6 14.8 15 15.215.4

Acquisition Time (min)

**b**

**a**

**Figure 4 Multiple-reaction monitoring (a,b) and mass spectrum (c) of oleanolic acid in the sample**

13.744 min.

#


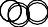

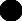

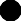

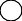

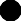

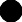


24-Ursolic acid - 8 Levels, 4 Levels Used, 12 Points, 5 Points Used, 0 QCs

x103 y = 11.347466 * x + 78.024346

R^2 = 0.99274113

2.5 Type:Linear, Origin:Ignore, Weight:1/x

2.4

2.3

2.2

2.1

2

1.9

1.8

1.7

1.6

1.5

1.4

1.3

1.2

1.1

1

0.9

0.8

0.7

0.6

0.5

0.4

0.3

0.2

0.1

0

-0.1

-0.2

-15 -10 -5 0 5 10 15 20 25 30 35 40 45 50 55 60 65 70 75 80 85 90 95 100 105 110 115 120 125 130 135 140 145 150 155 160 165 170 175 180 185 190 195 200 205 210 215

Concentration (ng/ml)

# Figure 5 Ursolic acid standard curve


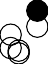

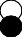

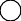

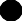

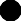

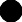


25-Oleanolic acid - 8 Levels, 5 Levels Used, 12 Points, 5 Points Used, 0 QCs

x103 y = 10.403155 * x + 248.035726

R^2 = 0.98742279

Type:Linear, Origin:Ignore, Weight:1/x

2.5

2.4

2.3

2.2

2.1

2

1.9

1.8

1.7

1.6

1.5

1.4

1.3

1.2

1.1

1

0.9

0.8

0.7

0.6

0.5

0.4

0.3

0.2

0.1

0

-0.1

-0.2

-15 -10 -5 0 5 10 15 20 25 30 35 40 45 50 55 60 65 70 75 80 85 90 95 100 105 110 115 120 125 130 135 140 145 150 155 160 165 170 175 180 185 190 195 200 205 210 215

Concentration (ng/ml)

# Figure 6 Oleanolic acid standard curve
