# Supplementary material for: Influence of geographic origin and tissue type on the medicinal chemical compounds of Semiliquidambar cathayensis
Source: PeerJ. 2023 Jun 6;11:e15484. doi: 10.7717/peerj.15484 (PMC10252815; doi:10.7717/peerj.15484)

# Quantitative Analysis Complete Report

Agilent Technologies

| | | | |
|---|---|---|---|
| **Batch Path** | D:\MassHunter\Data\WBR\2022\20220127-PA25\QuantResults\1.batch.bin | | |
| **Analysis Time** | 1/29/2022 11:06:08 AM | **Analyst Name** | admin-PC\admin |
| **Report Time** | 1/29/2022 11:08:57 AM | **Reporter Name** | admin |
| **Last Calib Update** | 1/29/2022 11:06:07 AM | **Batch State** | Processed |
| **Quant Batch Version** | B.07.01SP1 | **Quant Report Version** | B.07.01 SP1 |

| | | | |
|---|---|---|---|
| **Acq. Time** | 1/27/2022 8:35:49 PM | **Data File** | BLANK1.d |
| **Sample Type** | Blank | **Sample Name** | BLANK |
| **Dilution** | 1 | **Acq. Method** | 20220127-PA25.m |

## Sample Chromatogram

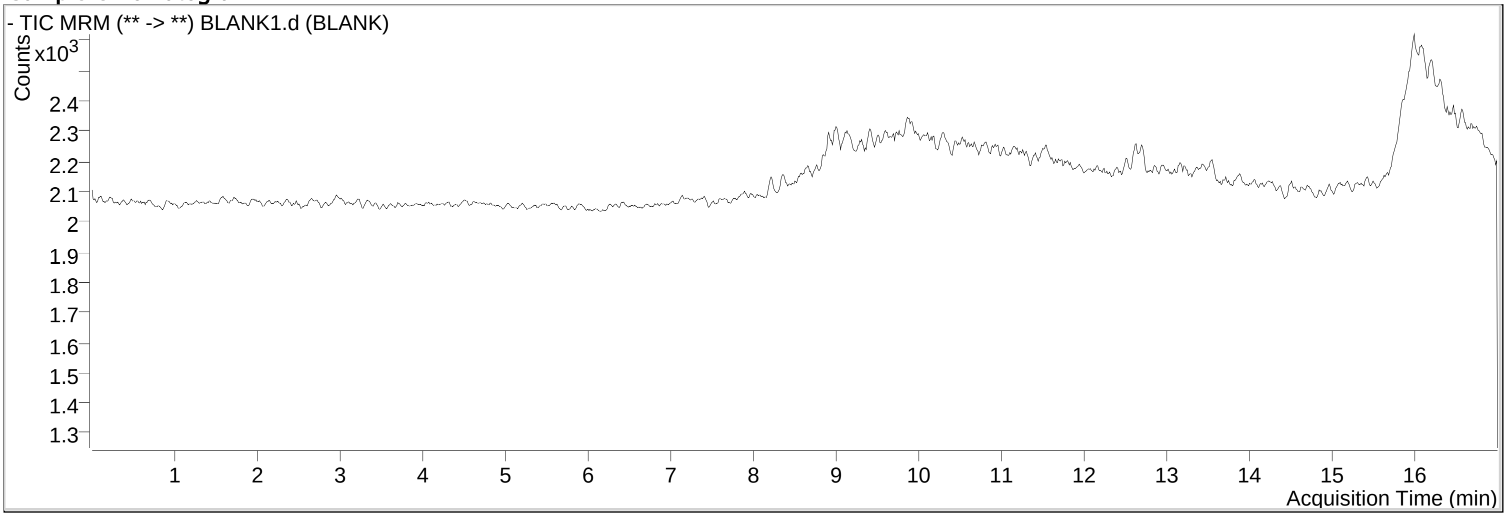


## 24-Ursolic acid

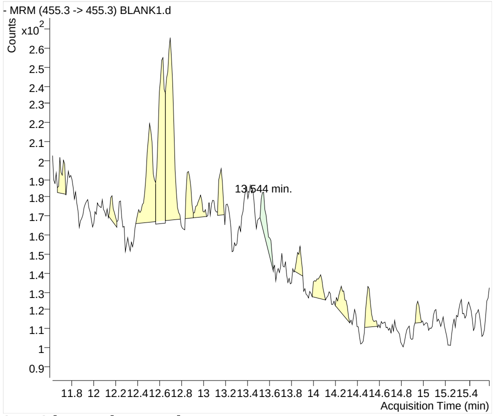


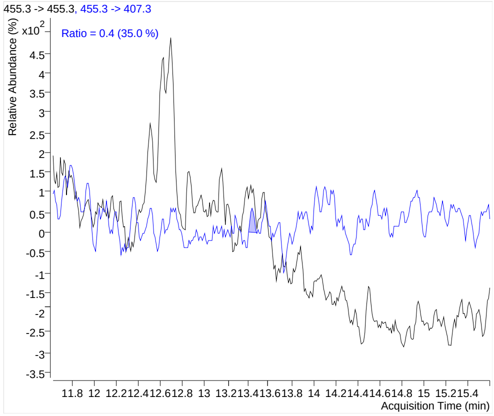


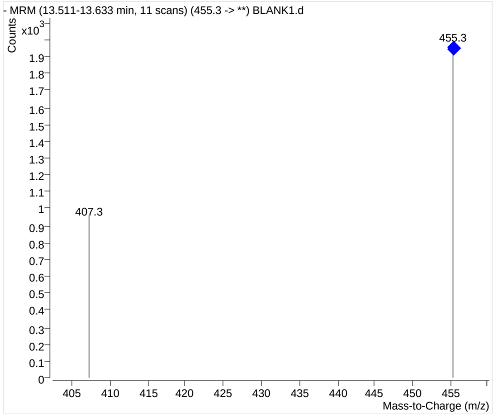


## 25-Oleanolic acid

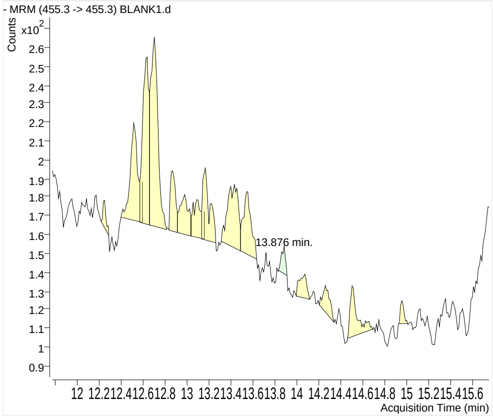


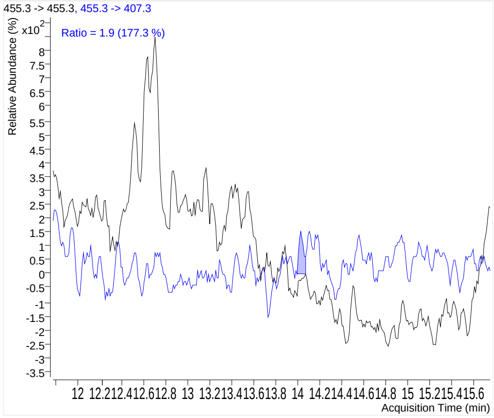


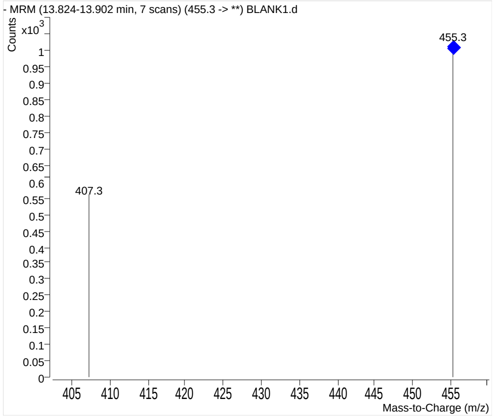

| | | | |
|---|---|---|---|
| **Batch Path** | D:\MassHunter\Data\WBR\2022\20220127-PA25\QuantResults\1.batch.bin | | |
| **Analysis Time** | 1/29/2022 11:06:08 AM | **Analyst Name** | admin-PC\admin |
| **Report Time** | 1/29/2022 11:09:01 AM | **Reporter Name** | admin |
| **Last Calib Update** | 1/29/2022 11:06:07 AM | **Batch State** | Processed |
| **Quant Batch Version** | B.07.01SP1 | **Quant Report Version** | B.07.01 SP1 |

| | | | |
|---|---|---|---|
| **Acq. Time** | 1/27/2022 8:53:31 PM | **Data File** | STD1-r001.d |
| **Sample Type** | Cal | **Sample Name** | STD1 |
| **Dilution** | 1 | **Acq. Method** | 20220127-PA25.m |

## Sample Chromatogram

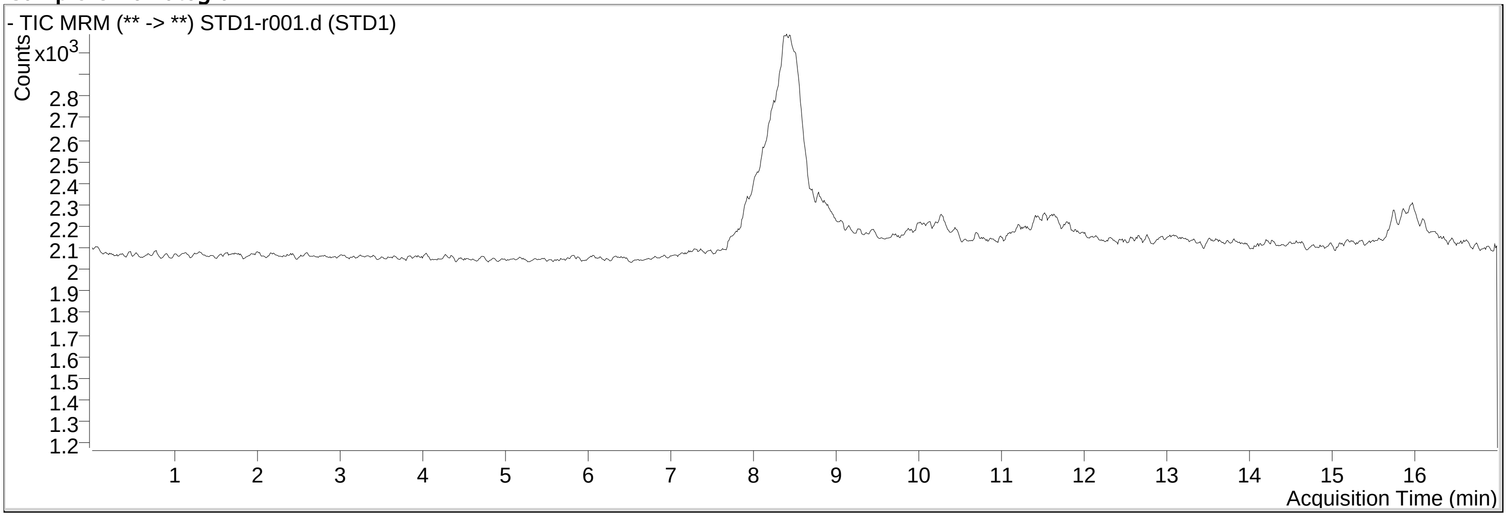


## 24-Ursolic acid

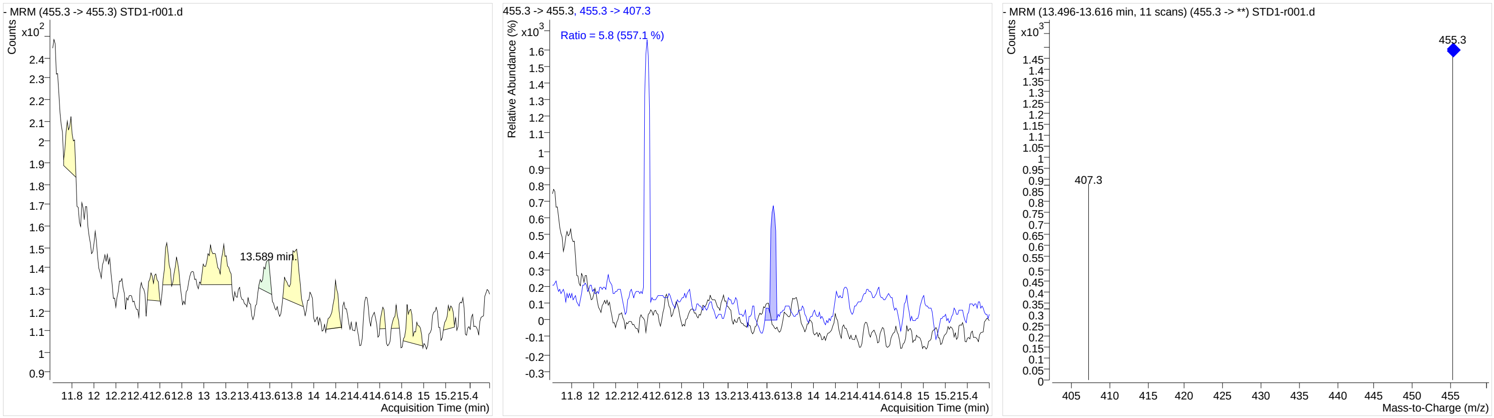


## 25-Oleanolic acid

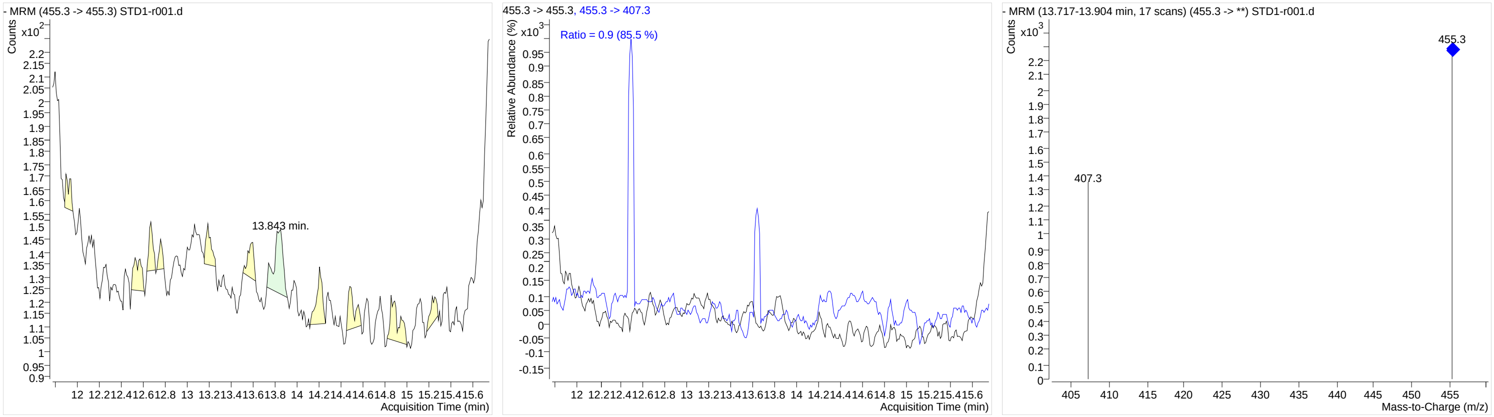

# Quantitative Analysis Complete Report

| | | | |
|---|---|---|---|
| **Batch Path** | D:\MassHunter\Data\WBR\2022\20220127-PA25\QuantResults\1.batch.bin | | |
| **Analysis Time** | 1/29/2022 11:06:08 AM | **Analyst Name** | admin-PC\admin |
| **Report Time** | 1/29/2022 11:09:05 AM | **Reporter Name** | admin |
| **Last Calib Update** | 1/29/2022 11:06:07 AM | **Batch State** | Processed |
| **Quant Batch Version** | B.07.01SP1 | **Quant Report Version** | B.07.01 SP1 |

| | | | |
|---|---|---|---|
| **Acq. Time** | 1/27/2022 9:11:14 PM | **Data File** | STD1-r002.d |
| **Sample Type** | Cal | **Sample Name** | STD1 |
| **Dilution** | 1 | **Acq. Method** | 20220127-PA25.m |

## Sample Chromatogram

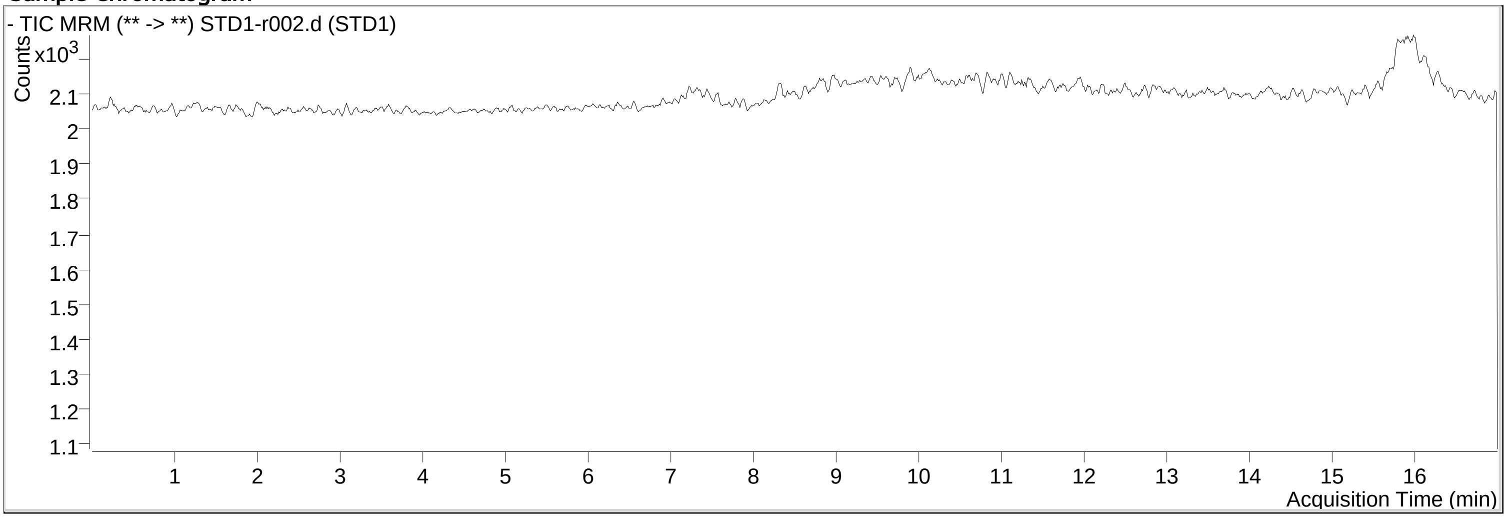


## 24-Ursolic acid

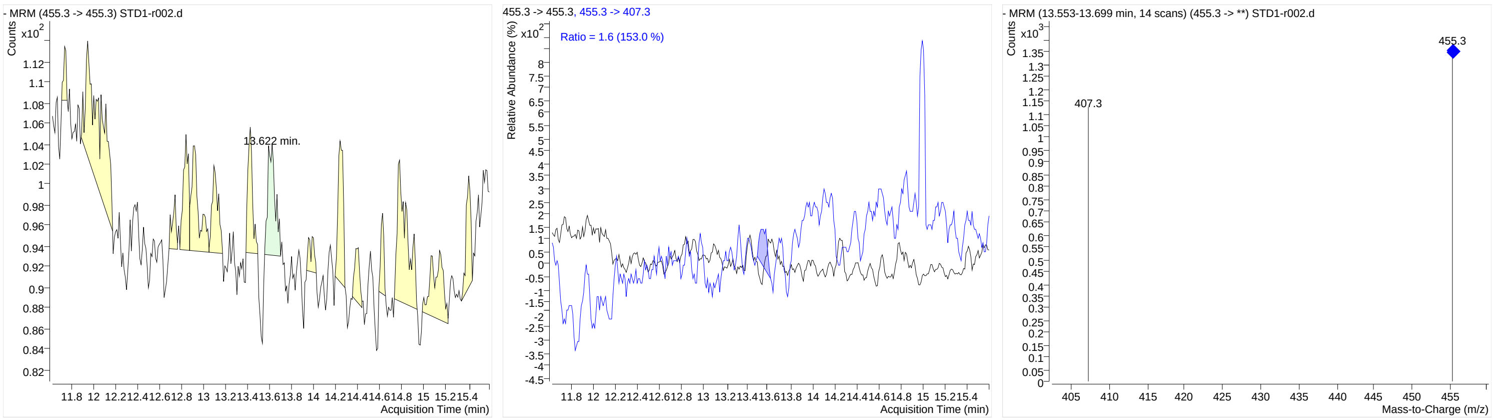


## 25-Oleanolic acid

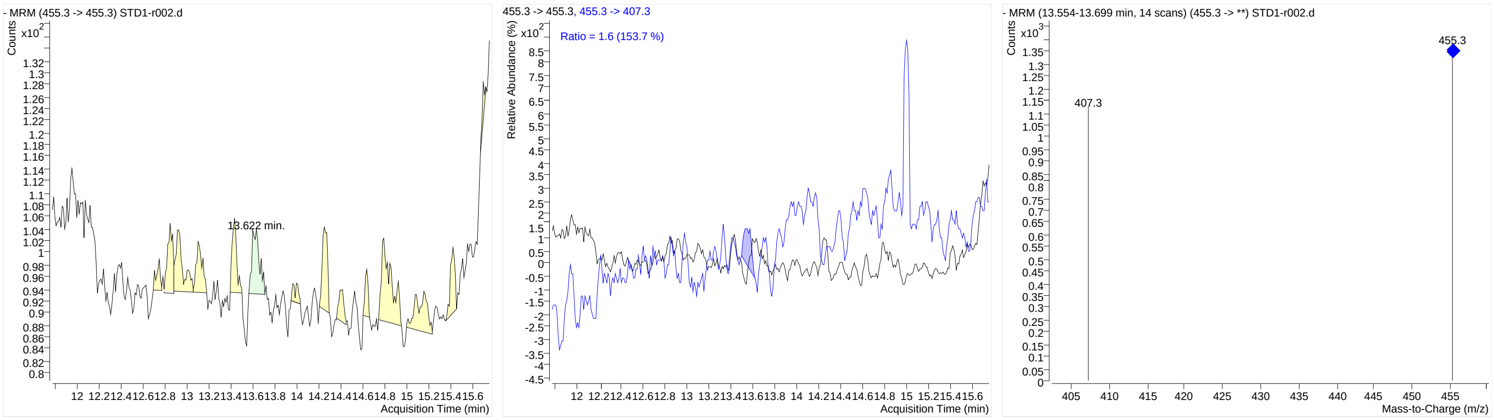

| | | | |
|---|---|---|---|
| **Batch Path** | D:\MassHunter\Data\WBR\2022\20220127-PA25\QuantResults\1.batch.bin | | |
| **Analysis Time** | 1/29/2022 11:06:08 AM | **Analyst Name** | admin-PC\admin |
| **Report Time** | 1/29/2022 11:09:09 AM | **Reporter Name** | admin |
| **Last Calib Update** | 1/29/2022 11:06:07 AM | **Batch State** | Processed |
| **Quant Batch Version** | B.07.01SP1 | **Quant Report Version** | B.07.01 SP1 |

| | | | |
|---|---|---|---|
| **Acq. Time** | 1/27/2022 9:28:55 PM | **Data File** | STD2-r001.d |
| **Sample Type** | Cal | **Sample Name** | STD2 |
| **Dilution** | 1 | **Acq. Method** | 20220127-PA25.m |

## Sample Chromatogram

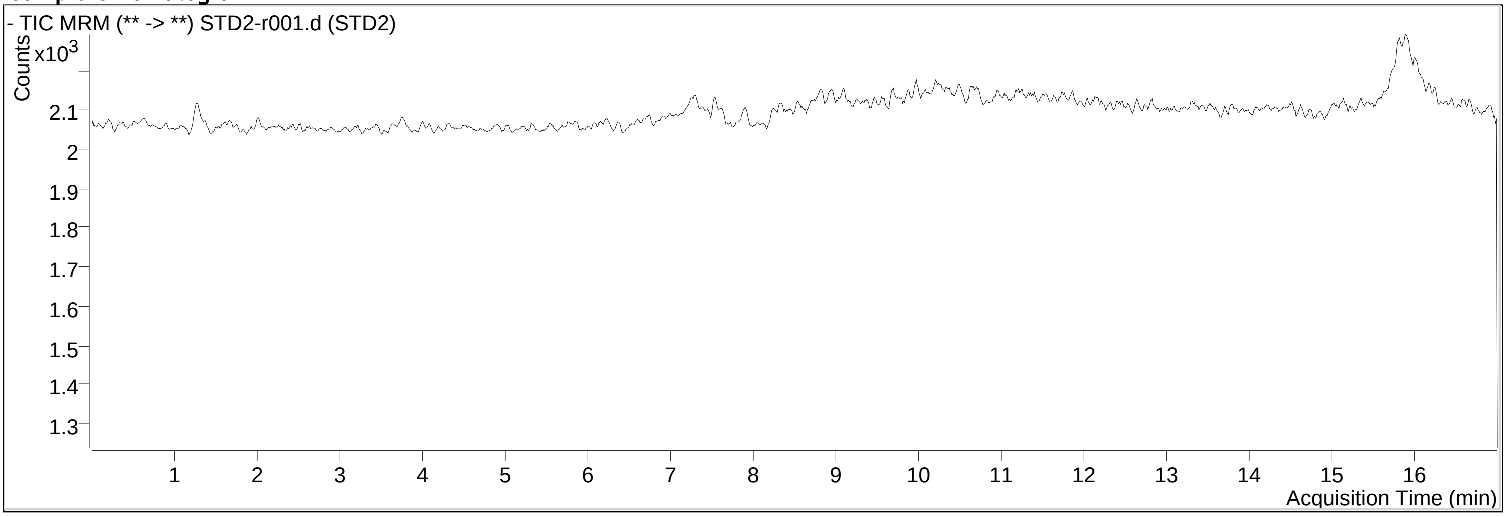


## 24-Ursolic acid

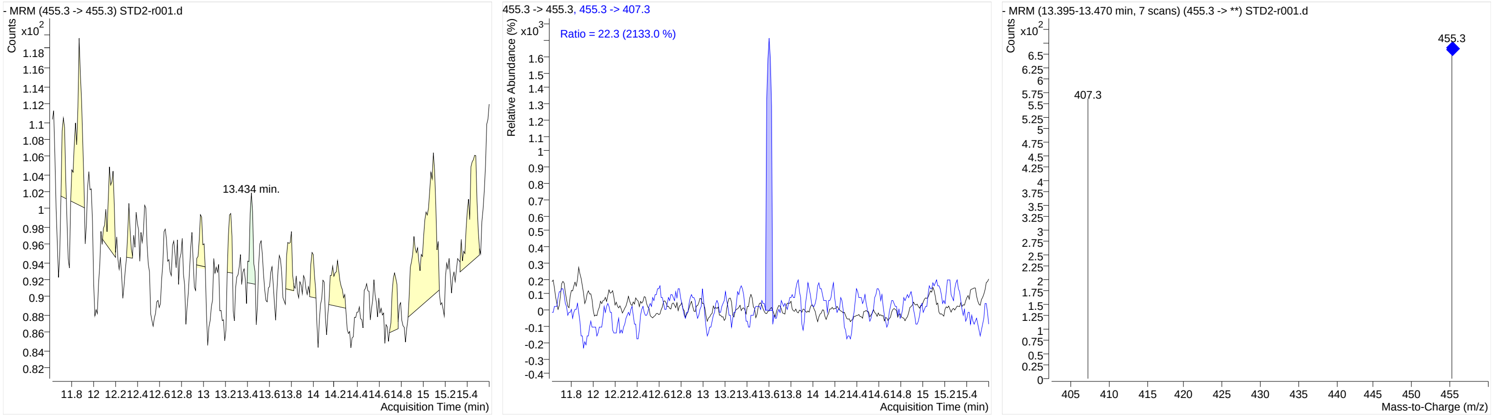


## 25-Oleanolic acid

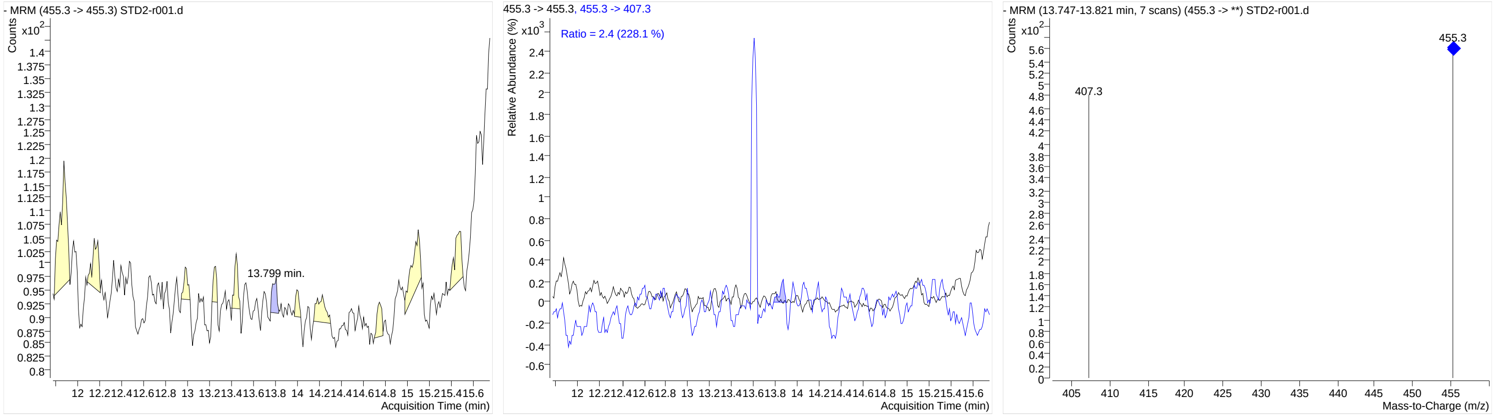

# Quantitative Analysis Complete Report

| | | | |
|---|---|---|---|
| **Batch Path** | D:\MassHunter\Data\WBR\2022\20220127-PA25\QuantResults\1.batch.bin | | |
| **Analysis Time** | 1/29/2022 11:06:08 AM | **Analyst Name** | admin-PC\admin |
| **Report Time** | 1/29/2022 11:09:12 AM | **Reporter Name** | admin |
| **Last Calib Update** | 1/29/2022 11:06:07 AM | **Batch State** | Processed |
| **Quant Batch Version** | B.07.01SP1 | **Quant Report Version** | B.07.01 SP1 |

| | | | |
|---|---|---|---|
| **Acq. Time** | 1/27/2022 9:46:38 PM | **Data File** | STD2-r002.d |
| **Sample Type** | Cal | **Sample Name** | STD2 |
| **Dilution** | 1 | **Acq. Method** | 20220127-PA25.m |

## Sample Chromatogram

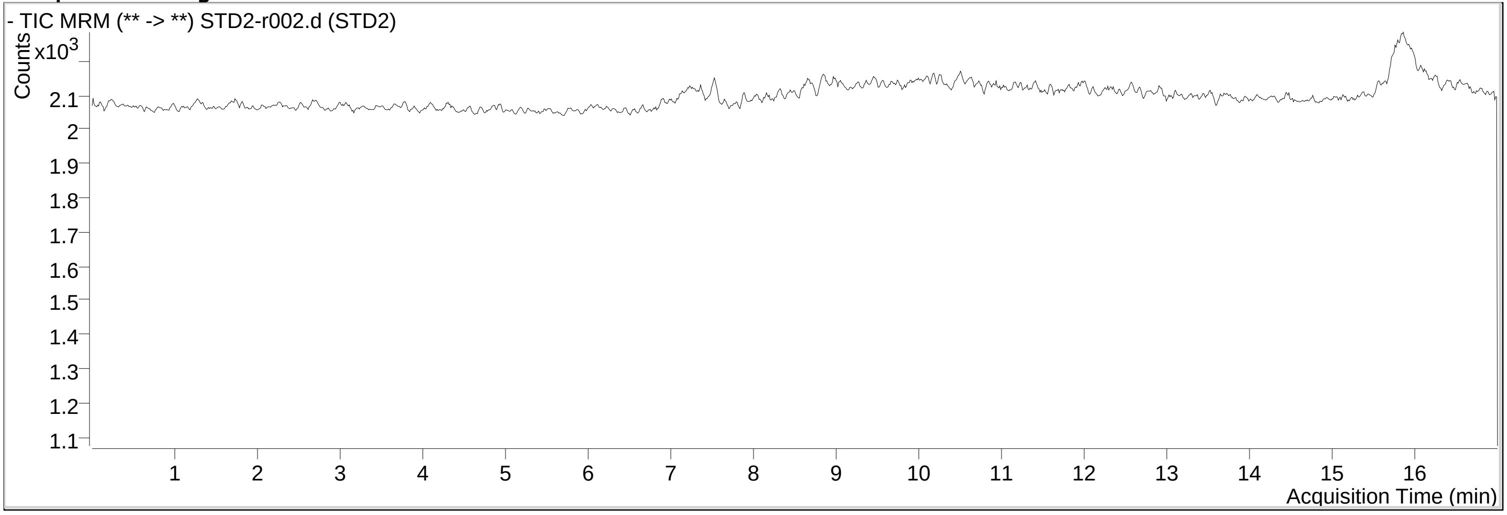


## 24-Ursolic acid

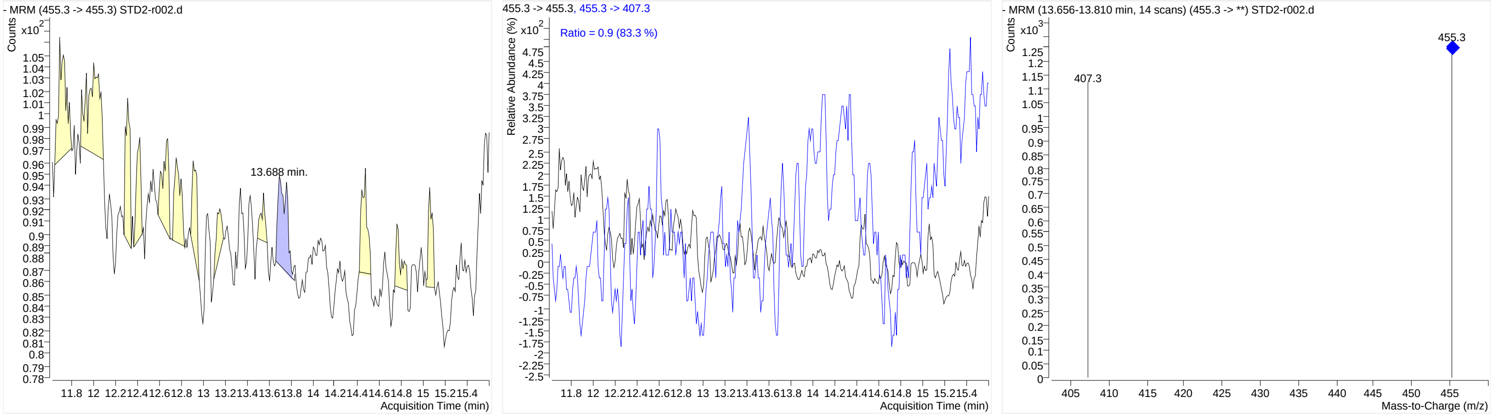


## 25-Oleanolic acid

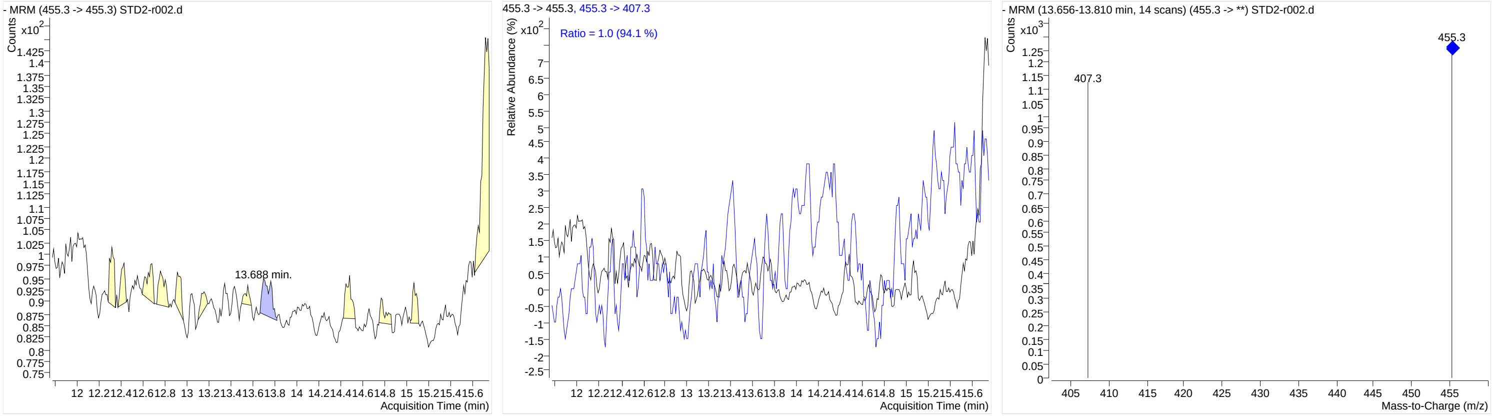

# Quantitative Analysis Complete Report

| | | | |
|---|---|---|---|
| **Batch Path** | D:\MassHunter\Data\WBR\2022\20220127-PA25\QuantResults\1.batch.bin | | |
| **Analysis Time** | 1/29/2022 11:06:08 AM | **Analyst Name** | admin-PC\admin |
| **Report Time** | 1/29/2022 11:09:16 AM | **Reporter Name** | admin |
| **Last Calib Update** | 1/29/2022 11:06:07 AM | **Batch State** | Processed |
| **Quant Batch Version** | B.07.01SP1 | **Quant Report Version** | B.07.01 SP1 |

| | | | |
|---|---|---|---|
| **Acq. Time** | 1/27/2022 10:04:19 PM | **Data File** | STD3-r001.d |
| **Sample Type** | Cal | **Sample Name** | STD3 |
| **Dilution** | 1 | **Acq. Method** | 20220127-PA25.m |

## Sample Chromatogram

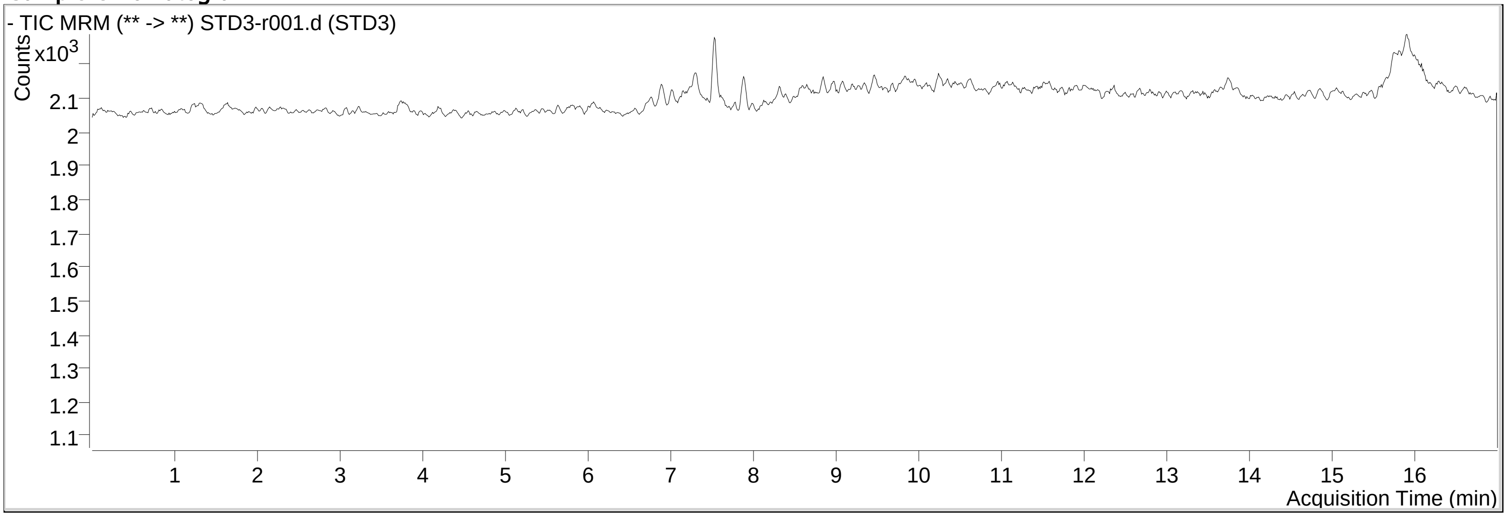


## 24-Ursolic acid

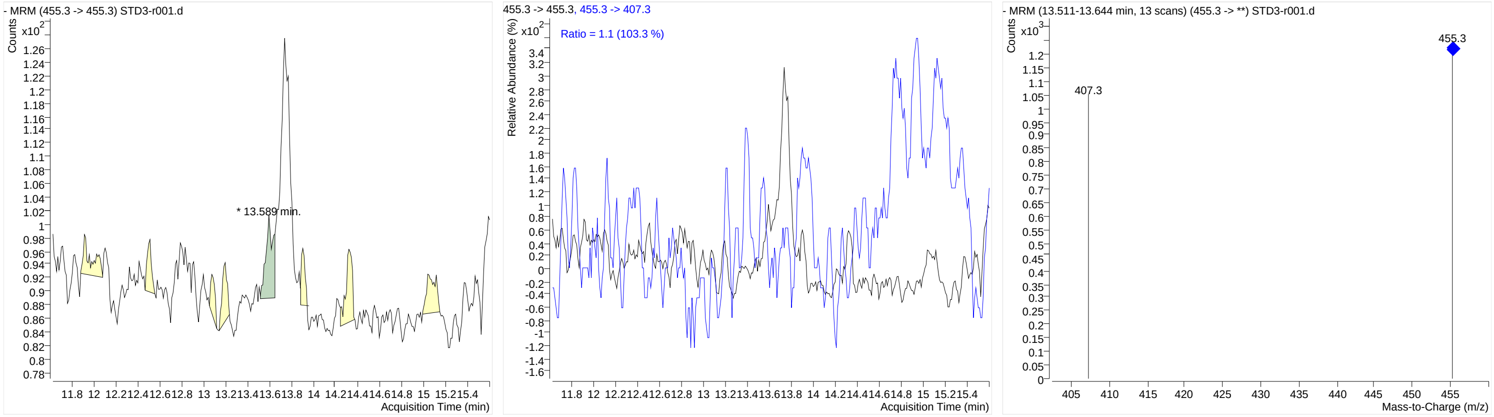


## 25-Oleanolic acid

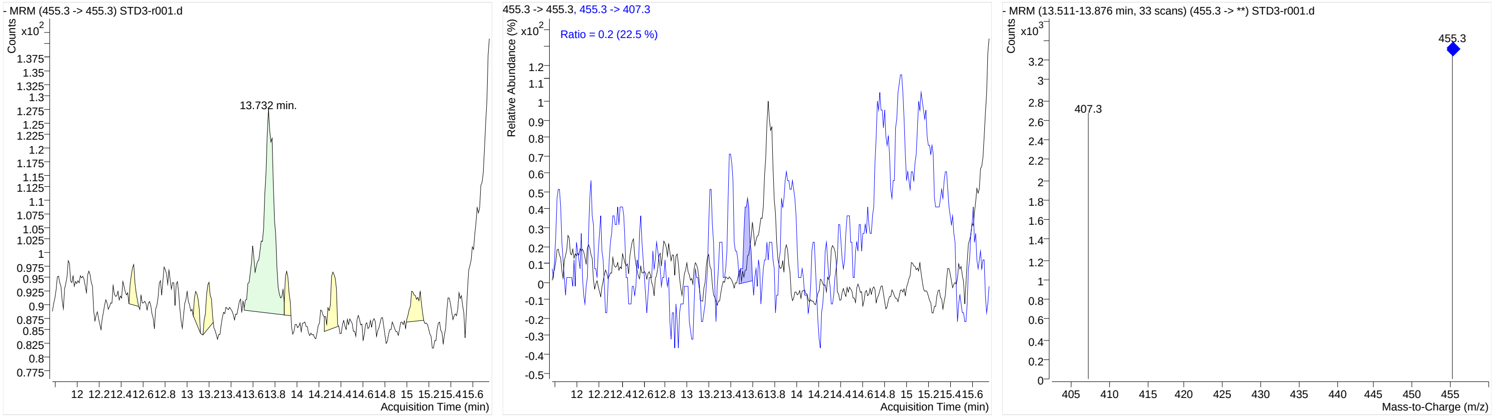

# Quantitative Analysis Complete Report

Agilent Technologies

| | | | |
|---|---|---|---|
| **Batch Path** | D:\MassHunter\Data\WBR\2022\20220127-PA25\QuantResults\1.batch.bin | | |
| **Analysis Time** | 1/29/2022 11:06:08 AM | **Analyst Name** | admin-PC\admin |
| **Report Time** | 1/29/2022 11:09:20 AM | **Reporter Name** | admin |
| **Last Calib Update** | 1/29/2022 11:06:07 AM | **Batch State** | Processed |
| **Quant Batch Version** | B.07.01SP1 | **Quant Report Version** | B.07.01 SP1 |

| | | | |
|---|---|---|---|
| **Acq. Time** | 1/27/2022 10:22:01 PM | **Data File** | STD3-r002.d |
| **Sample Type** | Cal | **Sample Name** | STD3 |
| **Dilution** | 1 | **Acq. Method** | 20220127-PA25.m |

## Sample Chromatogram

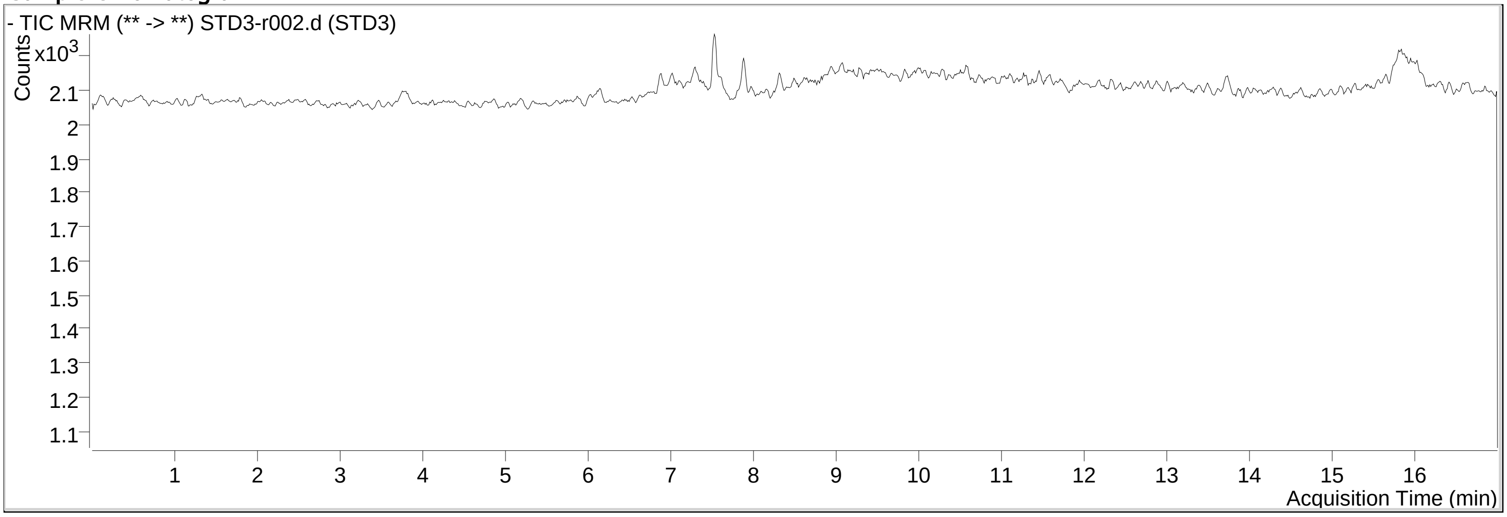


## 24-Ursolic acid

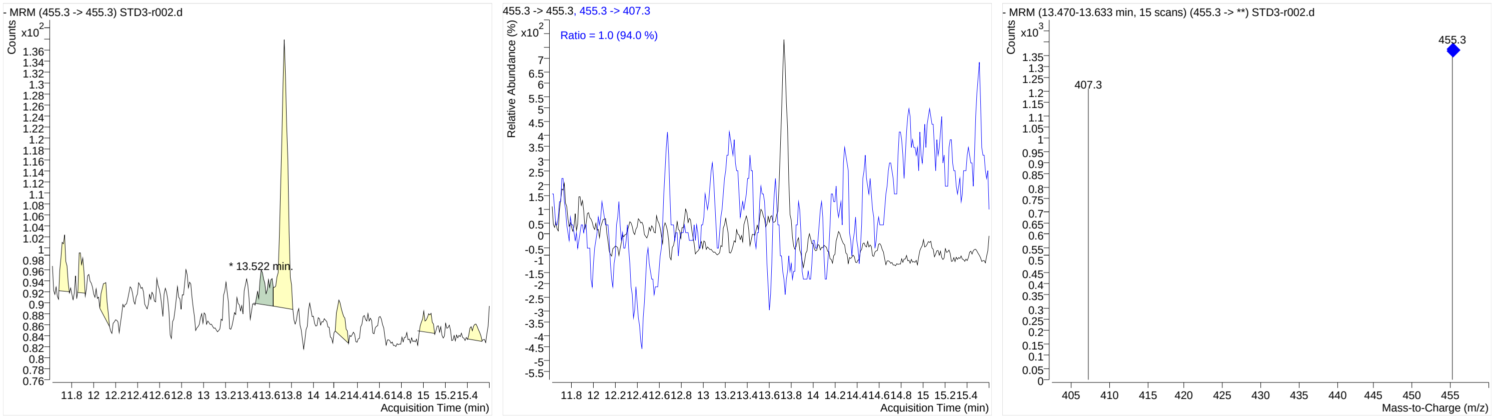


## 25-Oleanolic acid

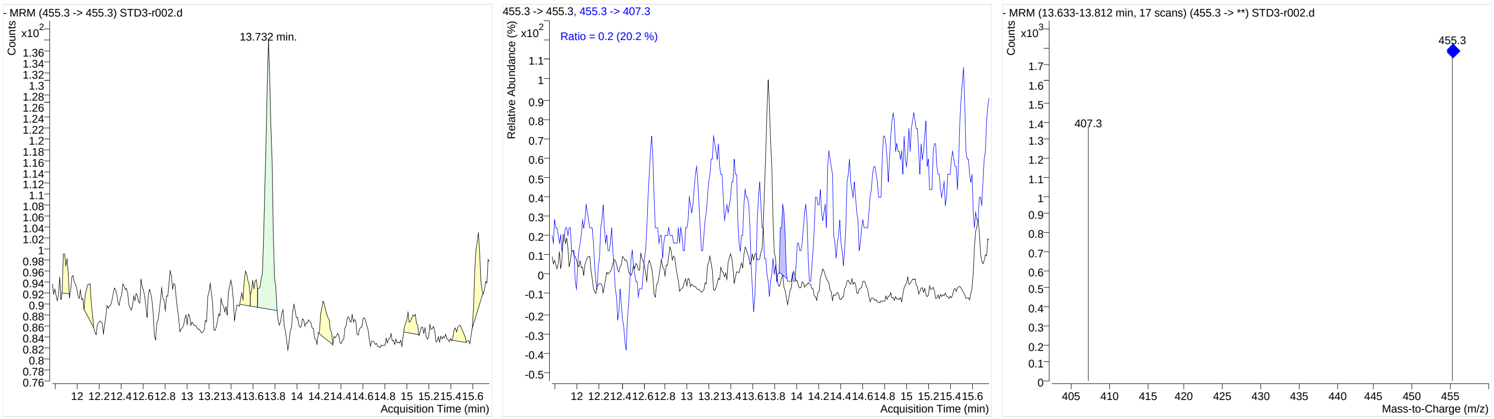

| | | | |
|---|---|---|---|
| Batch Path | D:\MassHunter\Data\WBR\2022\20220127-PA25\QuantResults\1.batch.bin | | |
| Analysis Time | 1/29/2022 11:06:08 AM | Analyst Name | admin-PC\admin |
| Report Time | 1/29/2022 11:09:23 AM | Reporter Name | admin |
| Last Calib Update | 1/29/2022 11:06:07 AM | Batch State | Processed |
| Quant Batch Version | B.07.01SP1 | Quant Report Version | B.07.01 SP1 |

| | | | |
|---|---|---|---|
| Acq. Time | 1/27/2022 10:39:42 PM | Data File | STD4-r001.d |
| Sample Type | Cal | Sample Name | STD4 |
| Dilution | 1 | Acq. Method | 20220127-PA25.m |

## Sample Chromatogram

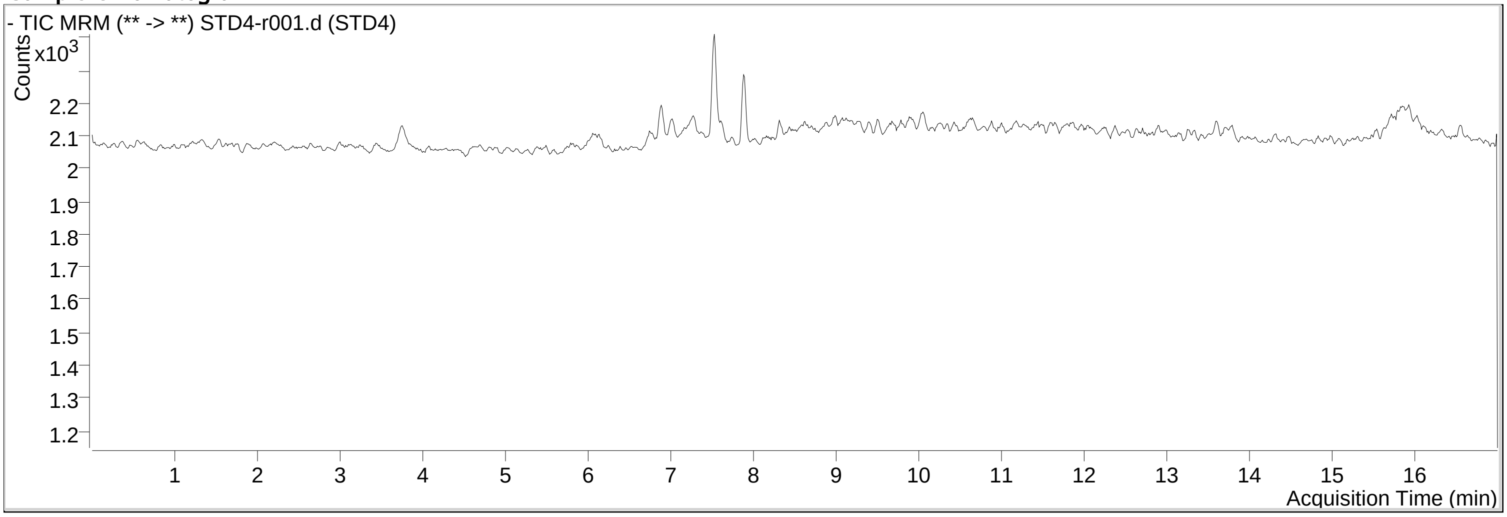


## 24-Ursolic acid

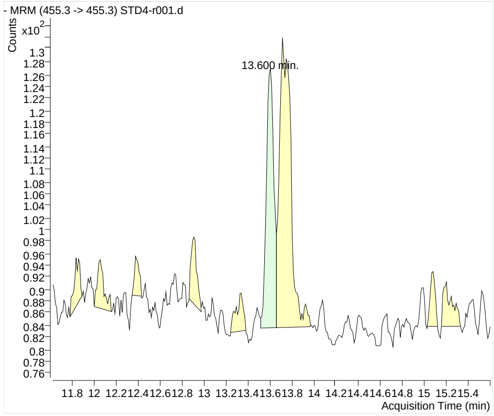


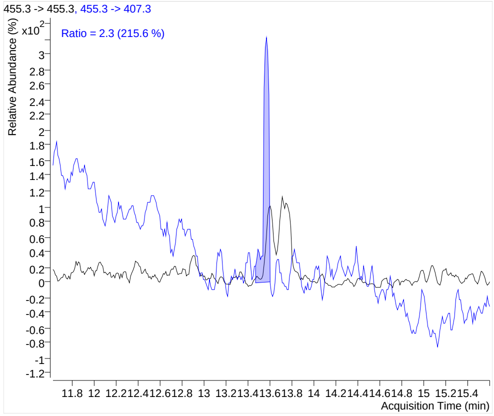


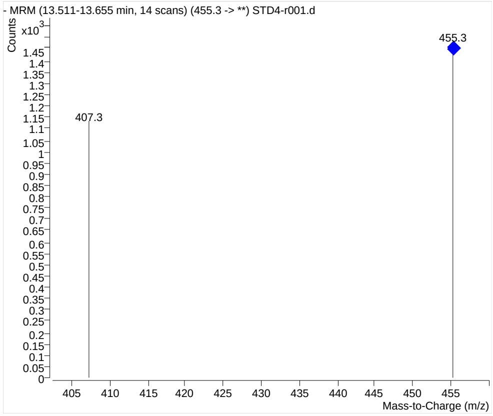


## 25-Oleanolic acid

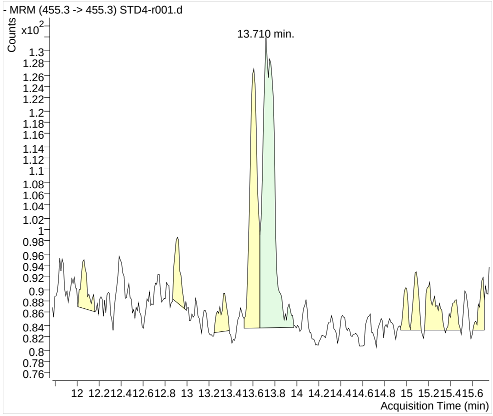


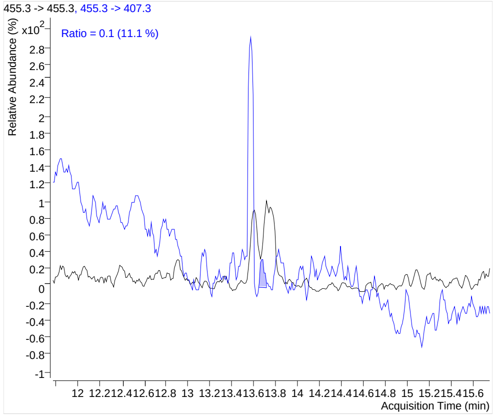


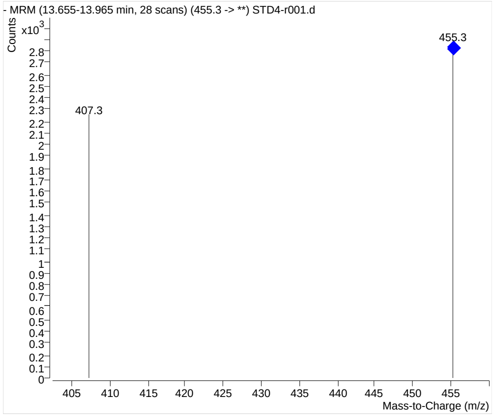

# Quantitative Analysis Complete Report

| | | | |
|---|---|---|---|
| **Batch Path** | D:\MassHunter\Data\WBR\2022\20220127-PA25\QuantResults\1.batch.bin | | |
| **Analysis Time** | 1/29/2022 11:06:08 AM | **Analyst Name** | admin-PC\admin |
| **Report Time** | 1/29/2022 11:09:27 AM | **Reporter Name** | admin |
| **Last Calib Update** | 1/29/2022 11:06:07 AM | **Batch State** | Processed |
| **Quant Batch Version** | B.07.01SP1 | **Quant Report Version** | B.07.01 SP1 |

| | | | |
|---|---|---|---|
| **Acq. Time** | 1/27/2022 10:57:24 PM | **Data File** | STD4-r002.d |
| **Sample Type** | Cal | **Sample Name** | STD4 |
| **Dilution** | 1 | **Acq. Method** | 20220127-PA25.m |

## Sample Chromatogram

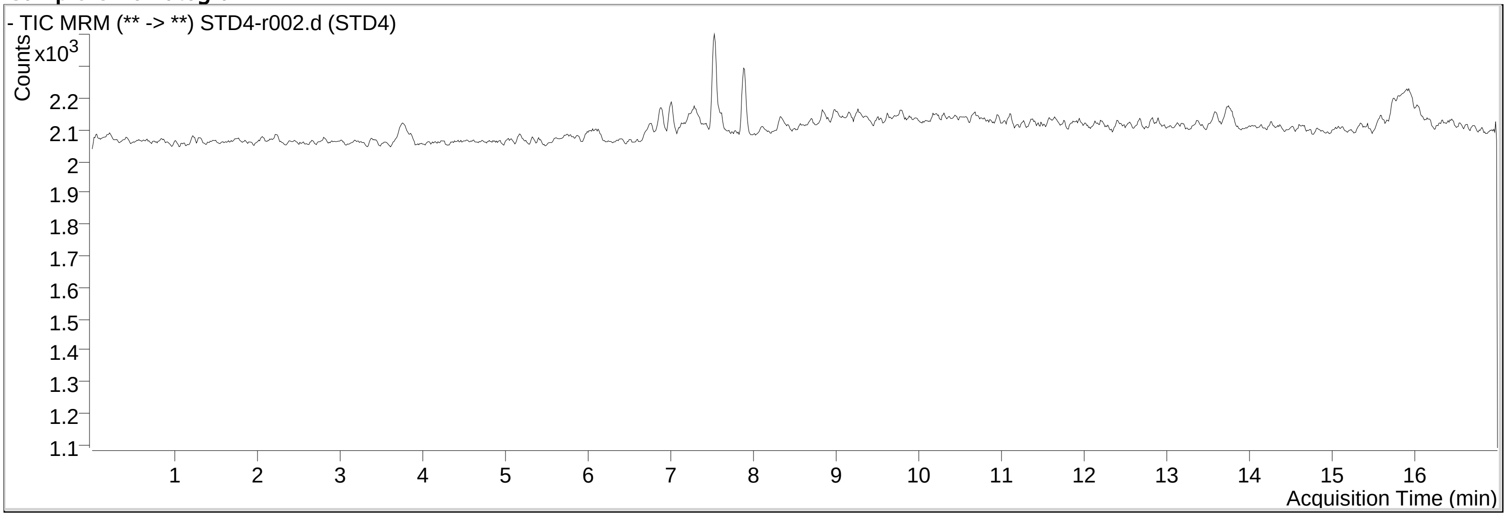


## 24-Ursolic acid

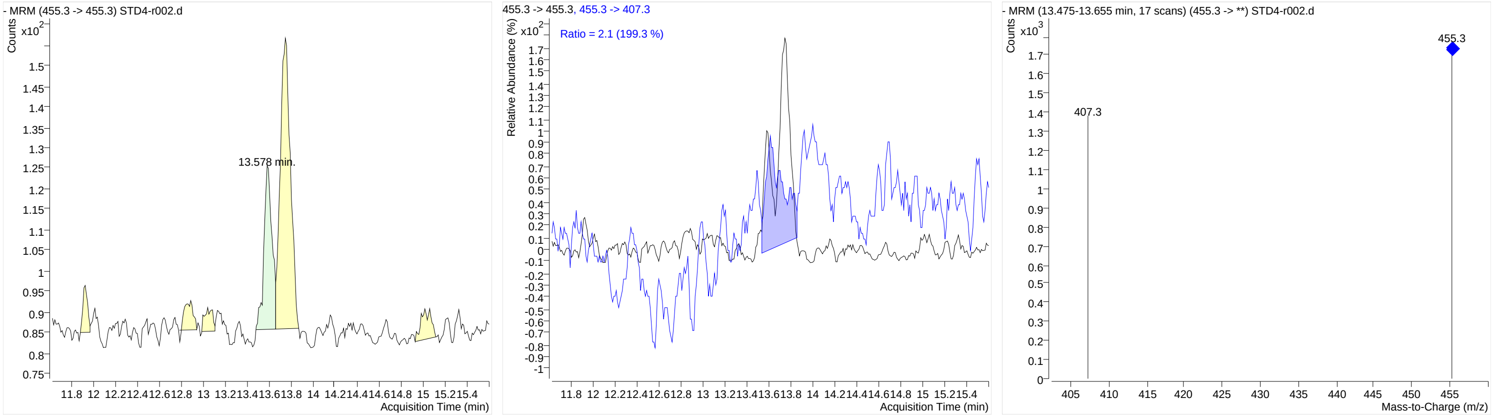


## 25-Oleanolic acid

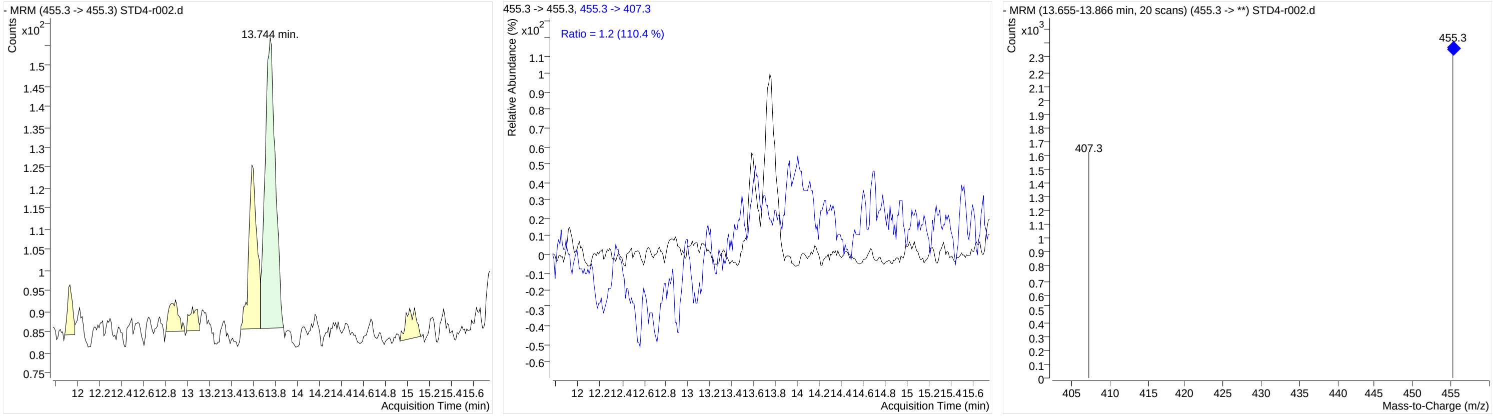

# Quantitative Analysis Complete Report

Agilent Technologies

| | | | |
|---|---|---|---|
| **Batch Path** | D:\MassHunter\Data\WBR\2022\20220127-PA25\QuantResults\1.batch.bin | | |
| **Analysis Time** | 1/29/2022 11:06:08 AM | **Analyst Name** | admin-PC\admin |
| **Report Time** | 1/29/2022 11:09:31 AM | **Reporter Name** | admin |
| **Last Calib Update** | 1/29/2022 11:06:07 AM | **Batch State** | Processed |
| **Quant Batch Version** | B.07.01SP1 | **Quant Report Version** | B.07.01 SP1 |

| | | | |
|---|---|---|---|
| **Acq. Time** | 1/27/2022 11:15:04 PM | **Data File** | STD5.d |
| **Sample Type** | Cal | **Sample Name** | STD5 |
| **Dilution** | 1 | **Acq. Method** | 20220127-PA25.m |

**Sample Chromatogram**

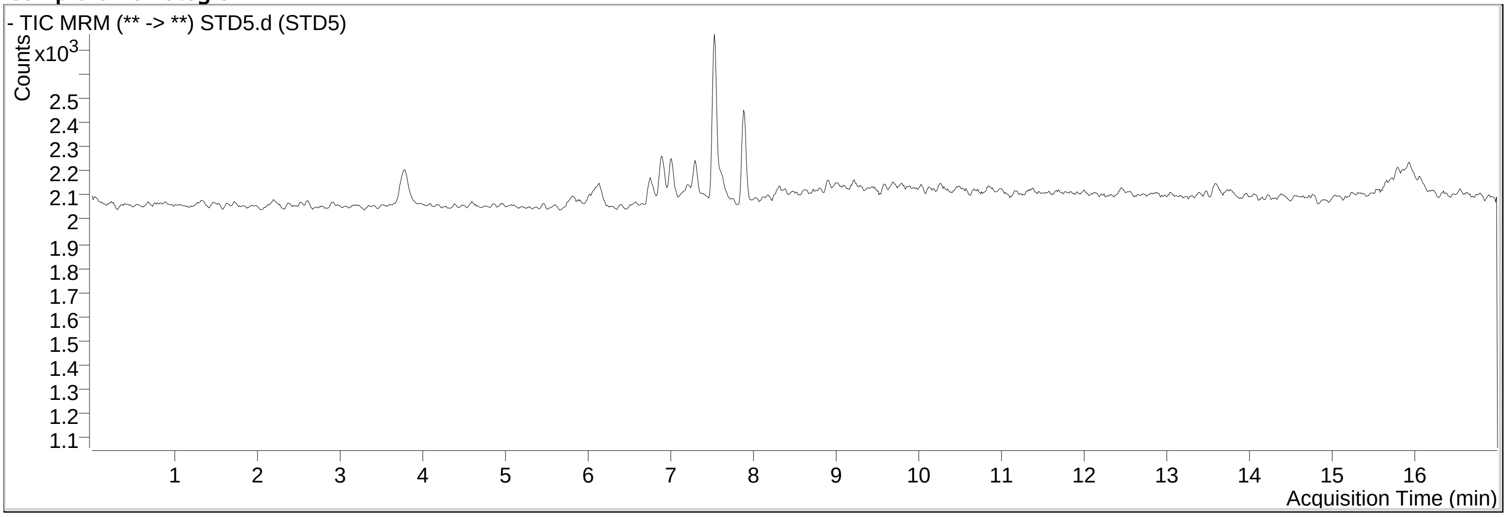


## 24-Ursolic acid

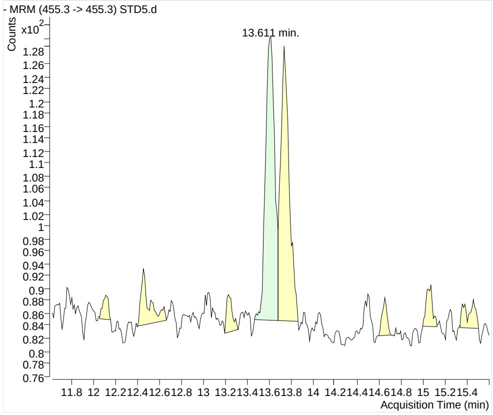


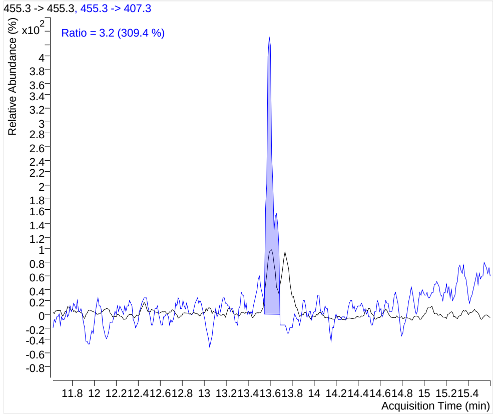


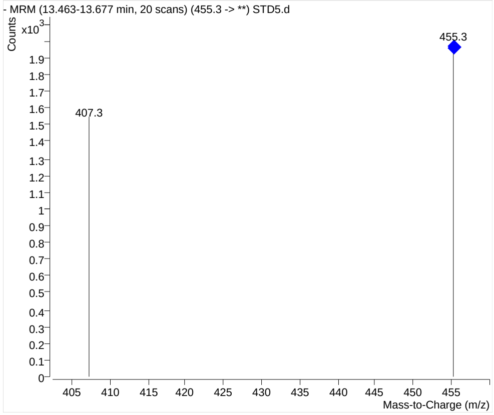


## 25-Oleanolic acid

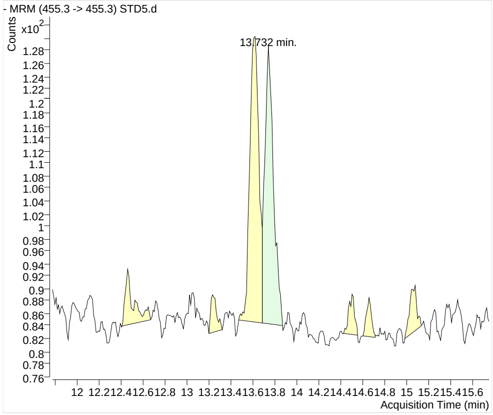


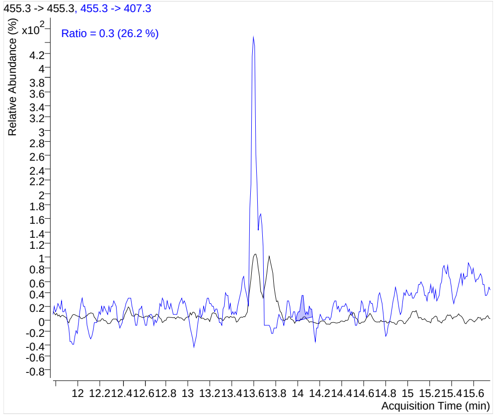


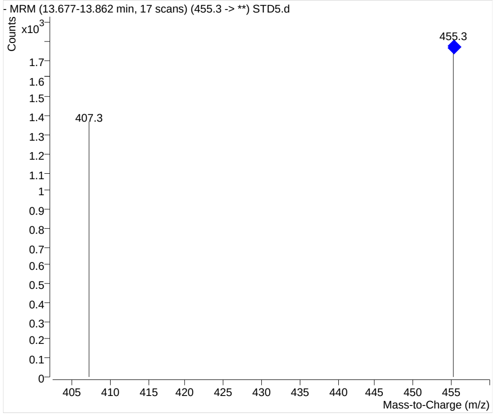

# Quantitative Analysis Complete Report

Agilent Technologies

| | | | |
|---|---|---|---|
| **Batch Path** | D:\MassHunter\Data\WBR\2022\20220127-PA25\QuantResults\1.batch.bin | | |
| **Analysis Time** | 1/29/2022 11:06:08 AM | **Analyst Name** | admin-PC\admin |
| **Report Time** | 1/29/2022 11:09:35 AM | **Reporter Name** | admin |
| **Last Calib Update** | 1/29/2022 11:06:07 AM | **Batch State** | Processed |
| **Quant Batch Version** | B.07.01SP1 | **Quant Report Version** | B.07.01 SP1 |

| | | | |
|---|---|---|---|
| **Acq. Time** | 1/27/2022 11:32:44 PM | **Data File** | STD6.d |
| **Sample Type** | Cal | **Sample Name** | STD6 |
| **Dilution** | 1 | **Acq. Method** | 20220127-PA25.m |

## Sample Chromatogram

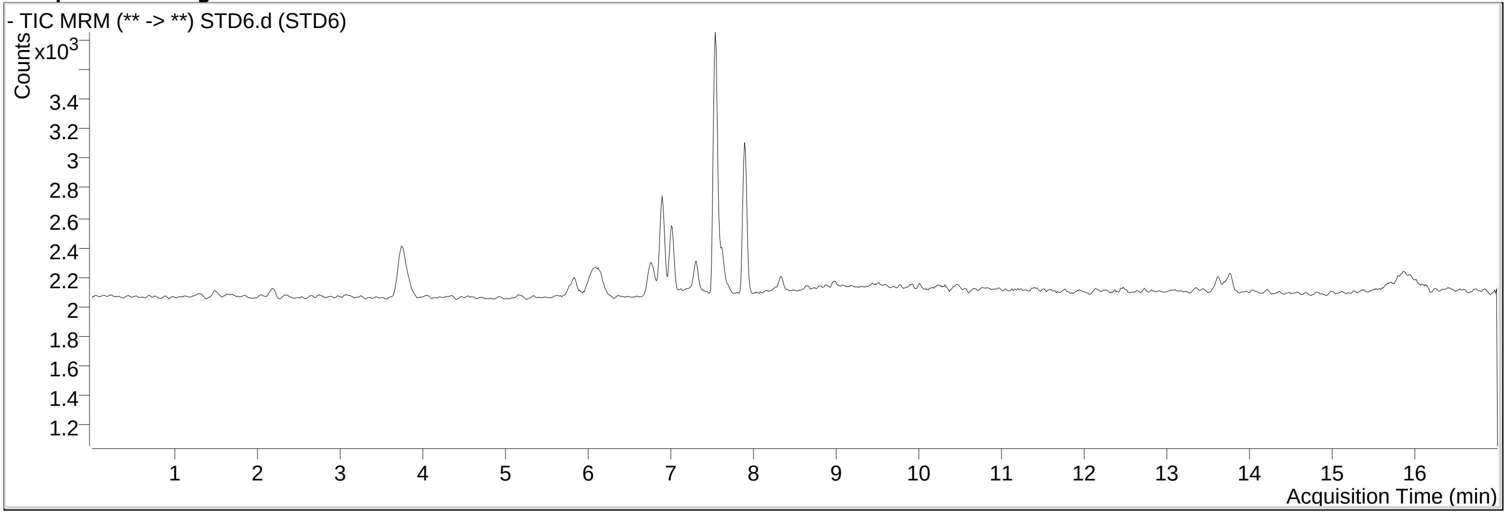


## 24-Ursolic acid

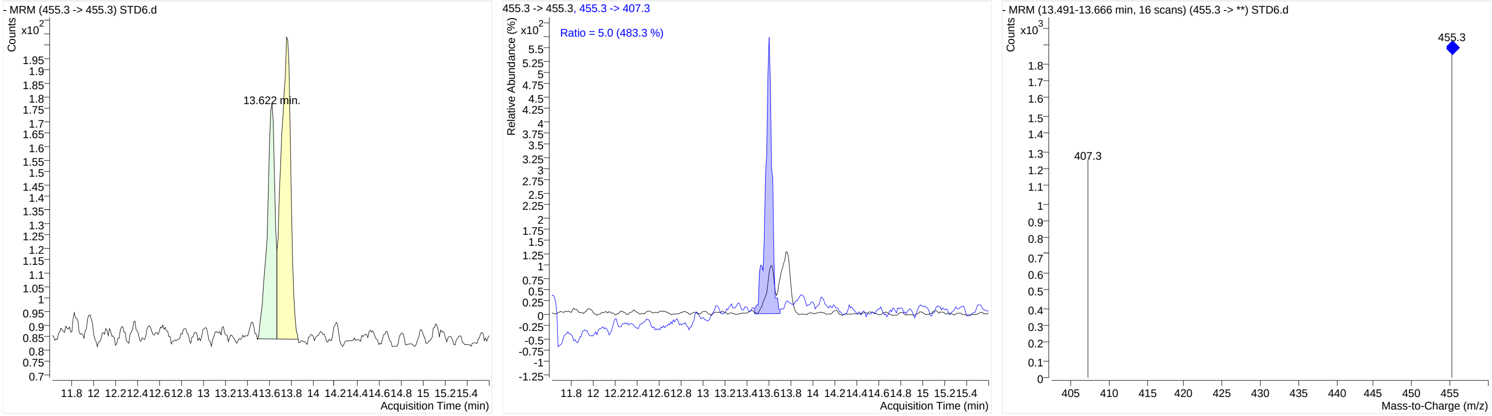


## 25-Oleanolic acid

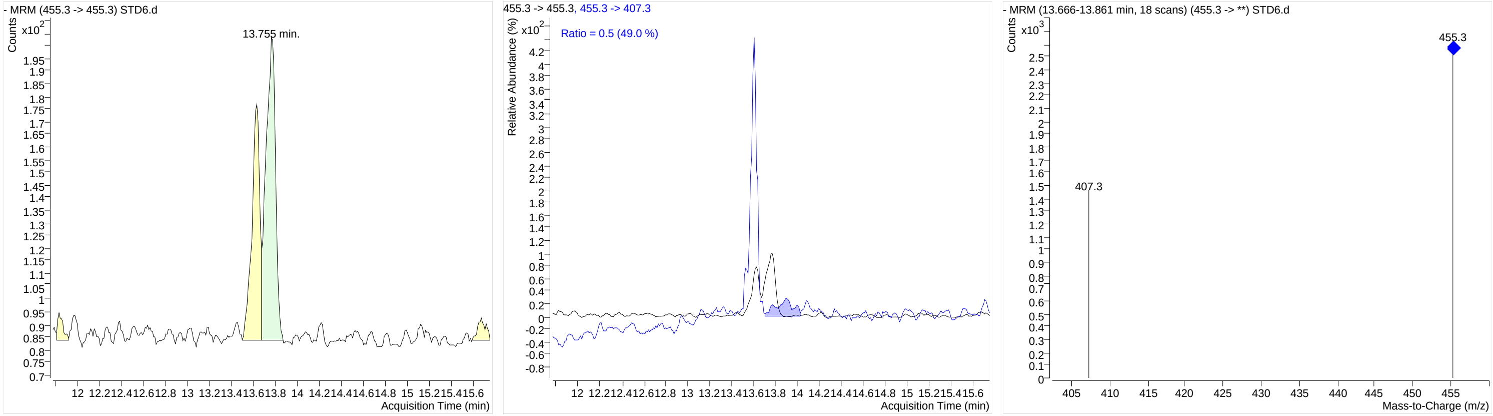

| | | | |
|---|---|---|---|
| **Batch Path** | D:\MassHunter\Data\WBR\2022\20220127-PA25\QuantResults\1.batch.bin | | |
| **Analysis Time** | 1/29/2022 11:06:08 AM | **Analyst Name** | admin-PC\admin |
| **Report Time** | 1/29/2022 11:09:38 AM | **Reporter Name** | admin |
| **Last Calib Update** | 1/29/2022 11:06:07 AM | **Batch State** | Processed |
| **Quant Batch Version** | B.07.01SP1 | **Quant Report Version** | B.07.01 SP1 |

| | | | |
|---|---|---|---|
| **Acq. Time** | 1/27/2022 11:50:24 PM | **Data File** | STD7.d |
| **Sample Type** | Cal | **Sample Name** | STD7 |
| **Dilution** | 1 | **Acq. Method** | 20220127-PA25.m |

## Sample Chromatogram

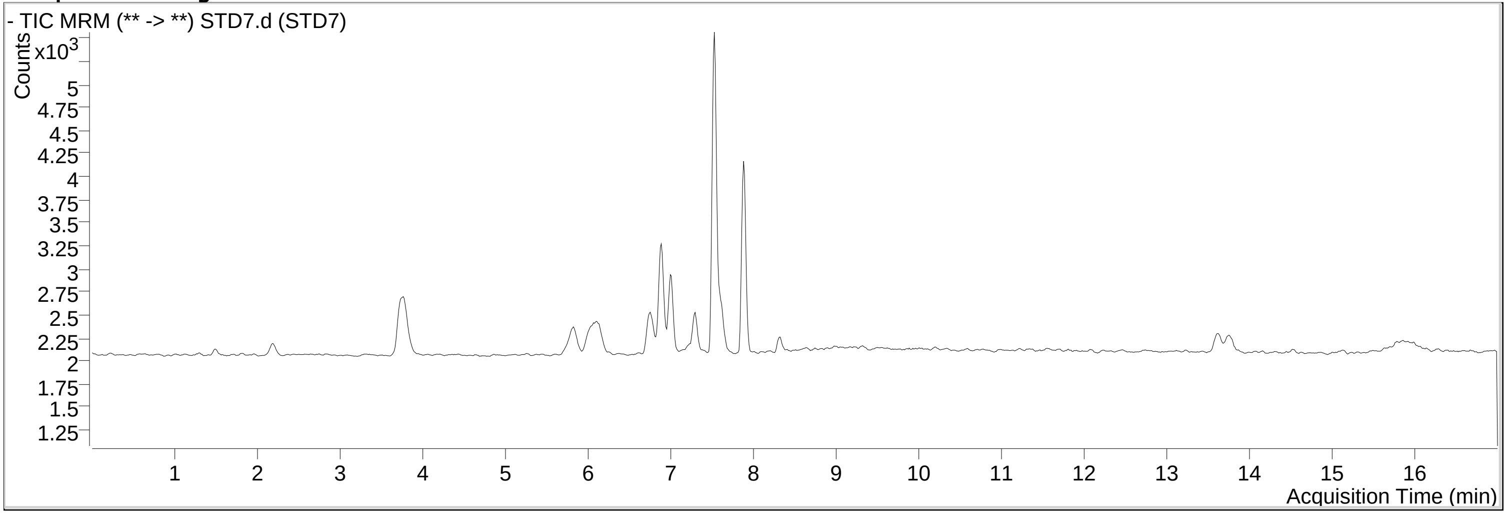


## 24-Ursolic acid

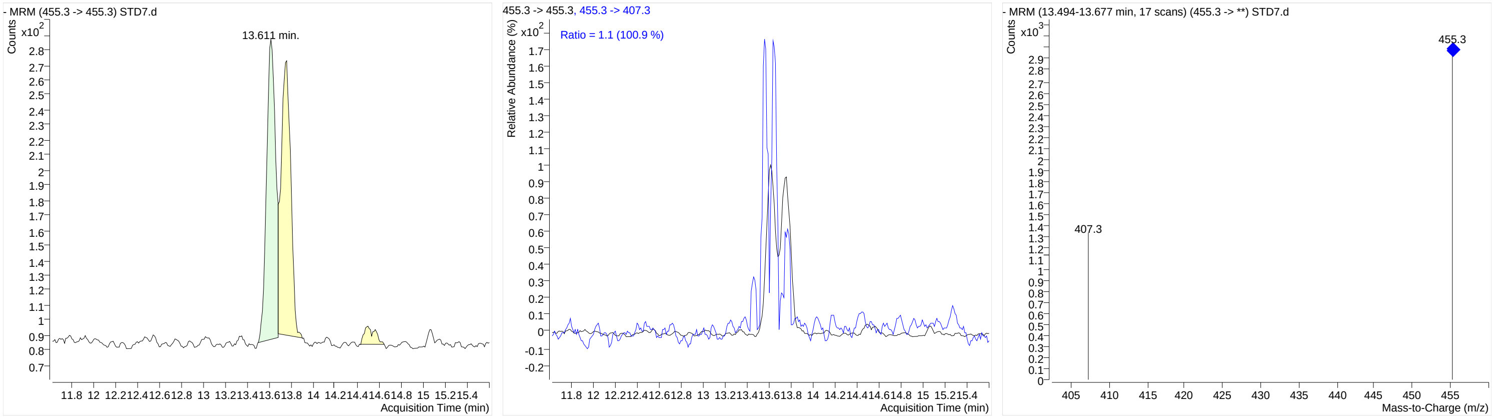


## 25-Oleanolic acid

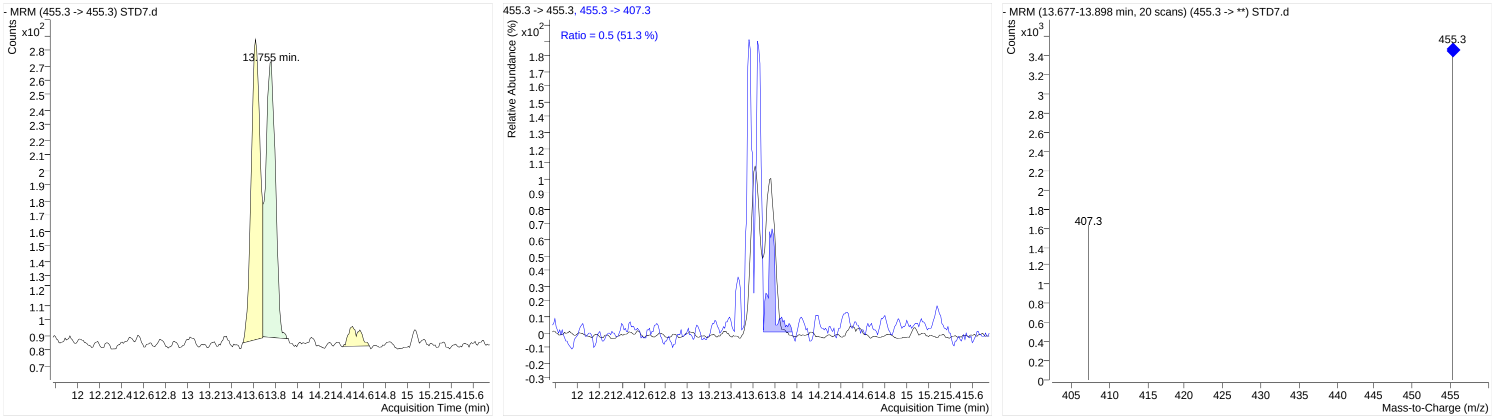

| | | | |
|---|---|---|---|
| **Batch Path** | D:\MassHunter\Data\WBR\2022\20220127-PA25\QuantResults\1.batch.bin | | |
| **Analysis Time** | 1/29/2022 11:06:08 AM | **Analyst Name** | admin-PC\admin |
| **Report Time** | 1/29/2022 11:09:42 AM | **Reporter Name** | admin |
| **Last Calib Update** | 1/29/2022 11:06:07 AM | **Batch State** | Processed |
| **Quant Batch Version** | B.07.01SP1 | **Quant Report Version** | B.07.01 SP1 |

| | | | |
|---|---|---|---|
| **Acq. Time** | 1/28/2022 12:08:05 AM | **Data File** | STD8.d |
| **Sample Type** | Cal | **Sample Name** | STD8 |
| **Dilution** | 1 | **Acq. Method** | 20220127-PA25.m |

## Sample Chromatogram

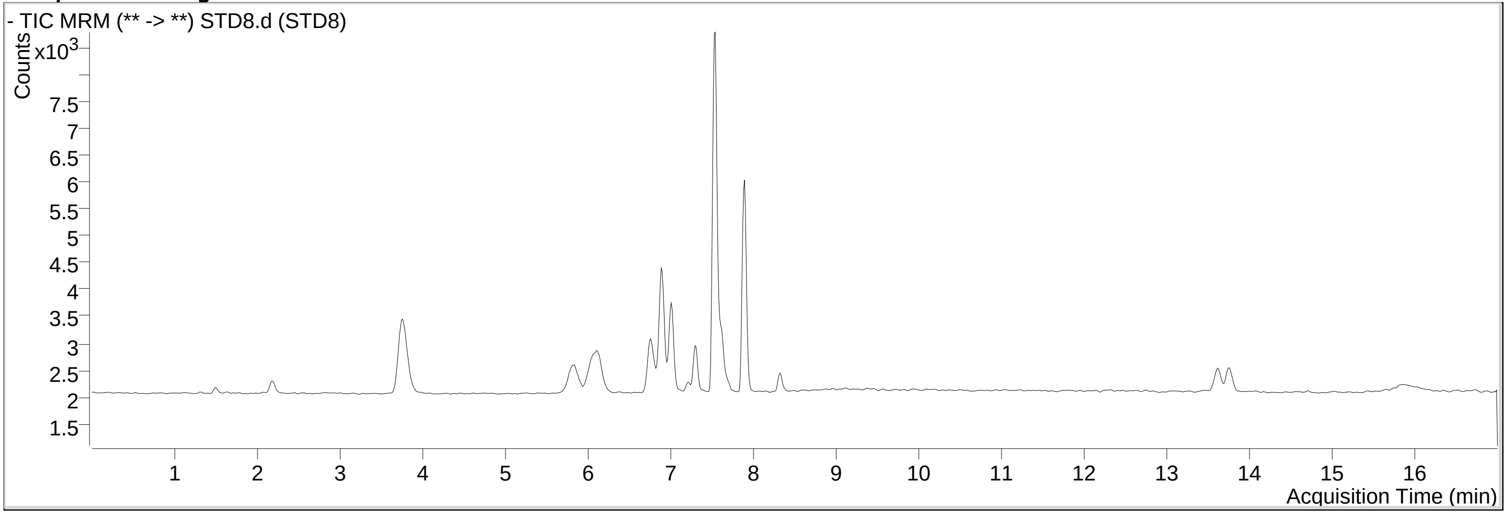


## 24-Ursolic acid

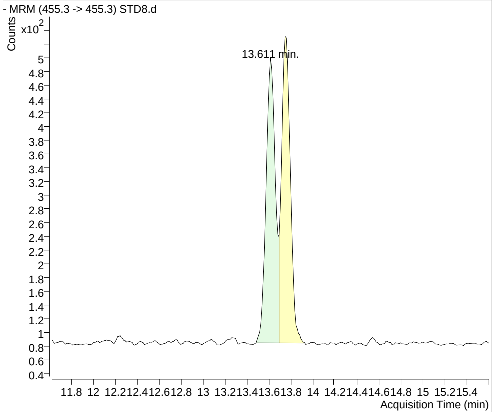


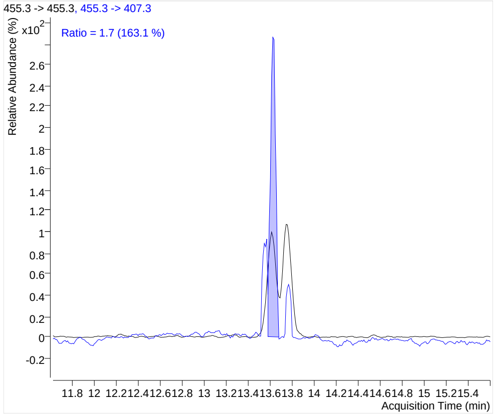


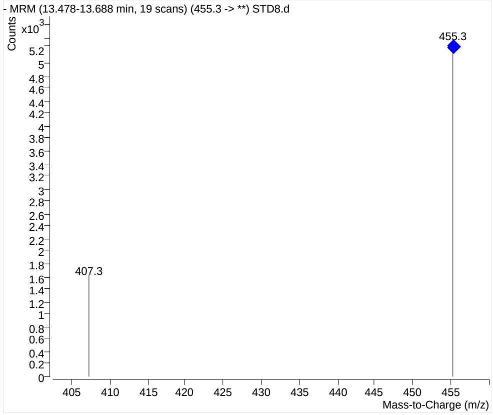


## 25-Oleanolic acid

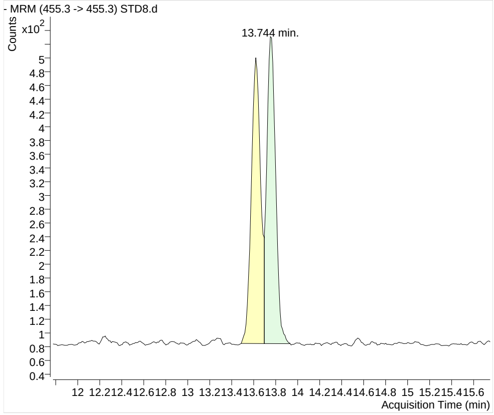


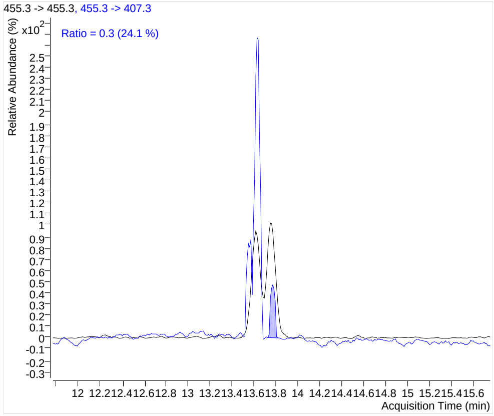


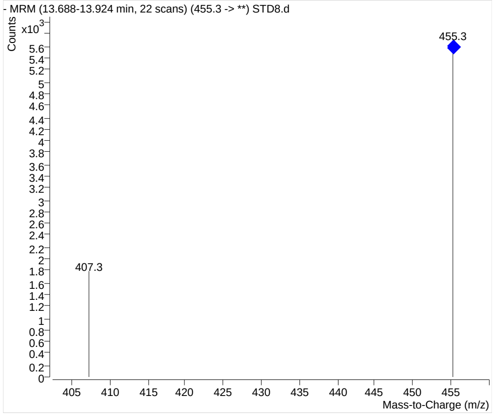

| | | | |
|---|---|---|---|
| **Batch Path** | D:\MassHunter\Data\WBR\2022\20220127-PA25\QuantResults\1.batch.bin | | |
| **Analysis Time** | 1/29/2022 11:06:08 AM | **Analyst Name** | admin-PC\admin |
| **Report Time** | 1/29/2022 11:09:49 AM | **Reporter Name** | admin |
| **Last Calib Update** | 1/29/2022 11:06:07 AM | **Batch State** | Processed |
| **Quant Batch Version** | B.07.01SP1 | **Quant Report Version** | B.07.01 SP1 |

| | | | |
|---|---|---|---|
| **Acq. Time** | 1/28/2022 12:43:31 AM | **Data File** | D1-r001.d |
| **Sample Type** | Sample | **Sample Name** | D1 |
| **Dilution** | 1 | **Acq. Method** | 20220127-PA25.m |

## Sample Chromatogram

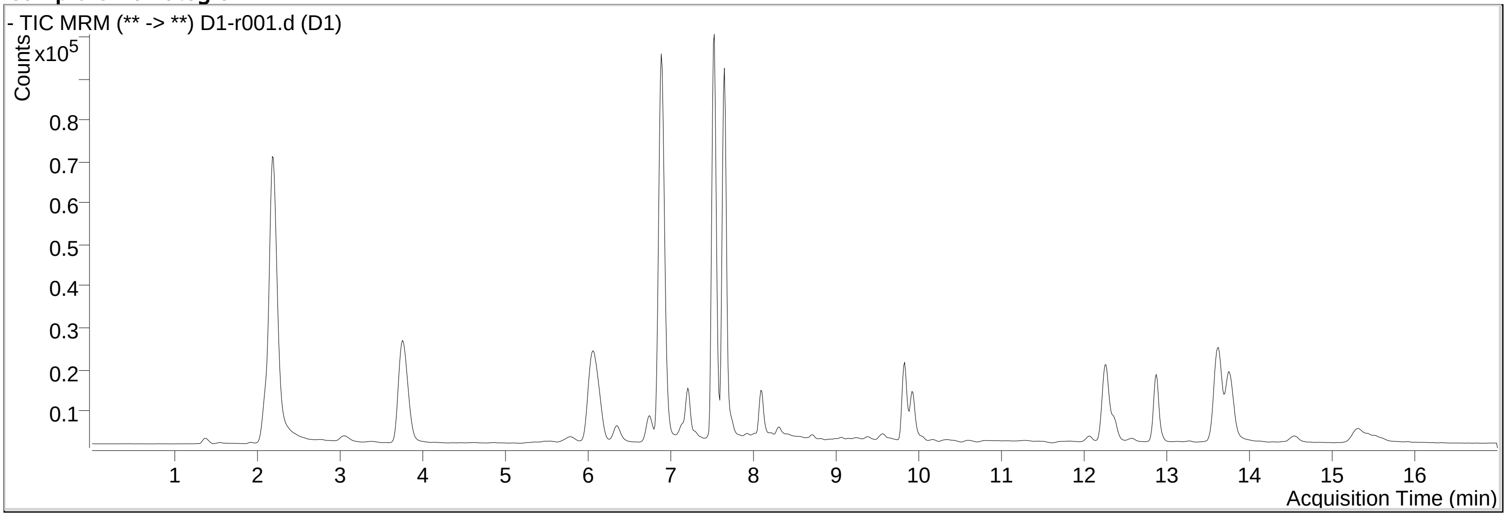


## 24-Ursolic acid

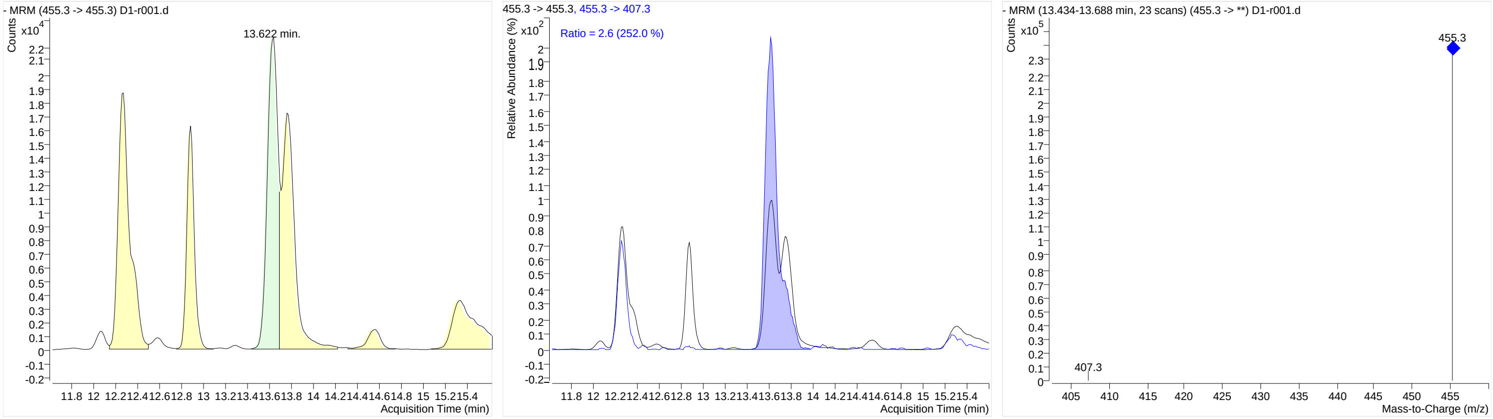


## 25-Oleanolic acid

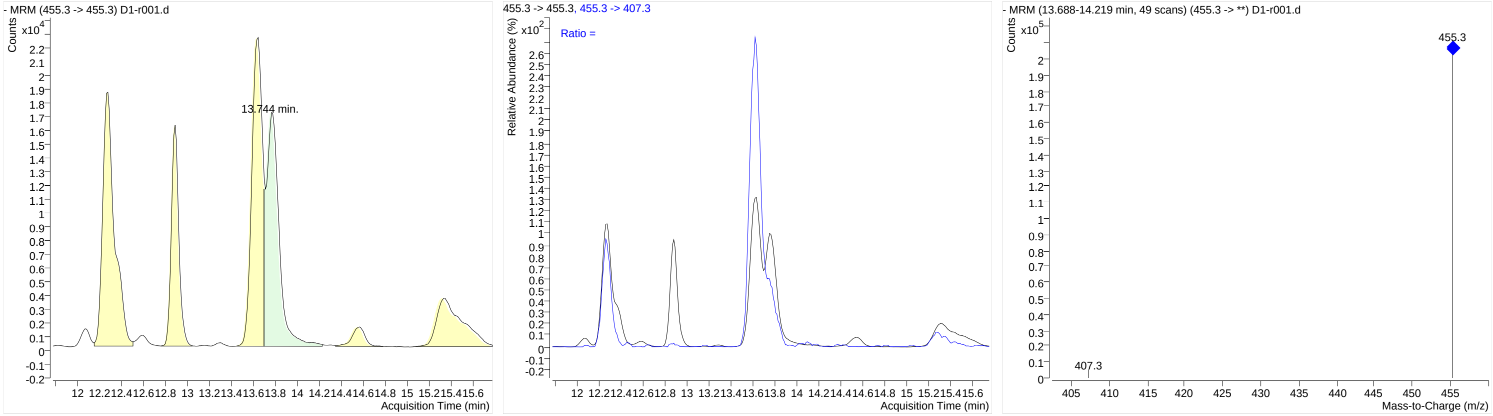

# Quantitative Analysis Complete Report

Agilent Technologies

| | | | |
|---|---|---|---|
| **Batch Path** | D:\MassHunter\Data\WBR\2022\20220127-PA25\QuantResults\1.batch.bin | | |
| **Analysis Time** | 1/29/2022 11:06:08 AM | **Analyst Name** | admin-PC\admin |
| **Report Time** | 1/29/2022 11:09:52 AM | **Reporter Name** | admin |
| **Last Calib Update** | 1/29/2022 11:06:07 AM | **Batch State** | Processed |
| **Quant Batch Version** | B.07.01SP1 | **Quant Report Version** | B.07.01 SP1 |

| | | | |
|---|---|---|---|
| **Acq. Time** | 1/28/2022 1:01:13 AM | **Data File** | D1-r002.d |
| **Sample Type** | Sample | **Sample Name** | D1 |
| **Dilution** | 1 | **Acq. Method** | 20220127-PA25.m |

## Sample Chromatogram

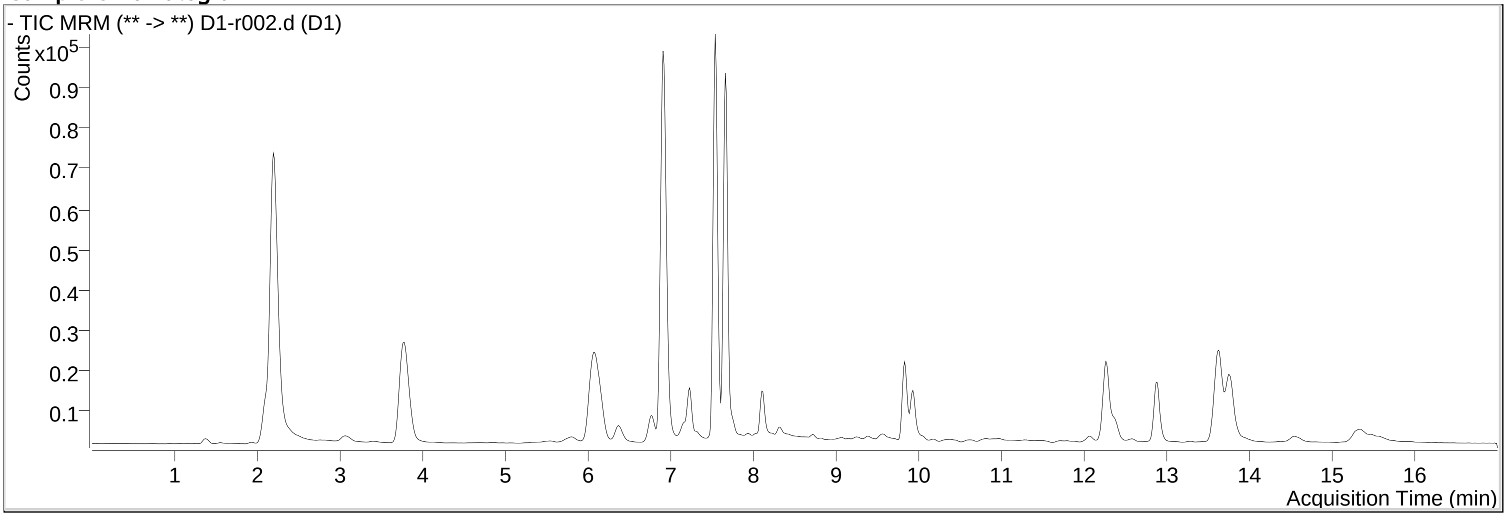


## 24-Ursolic acid

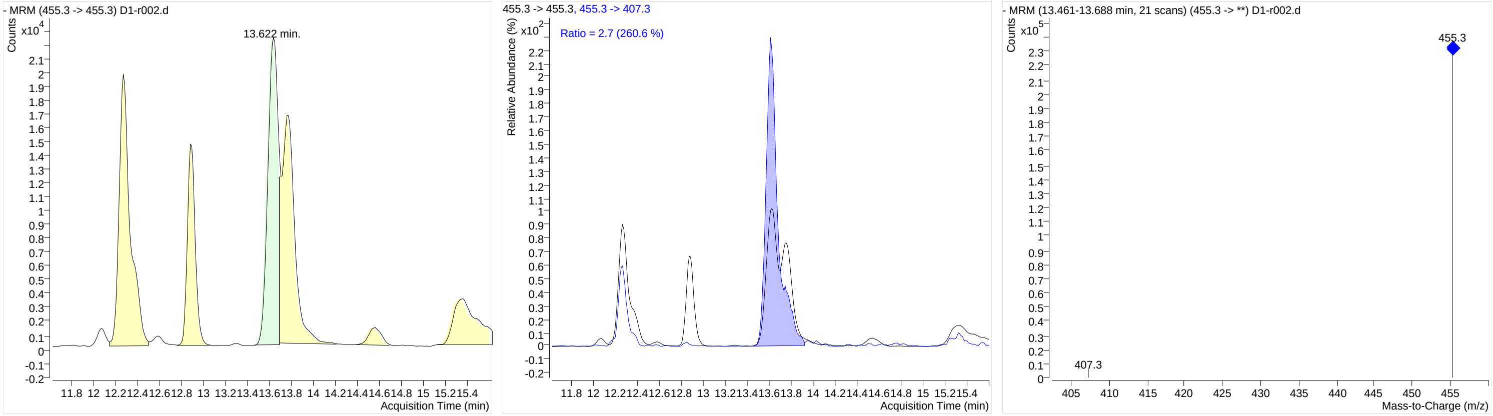


## 25-Oleanolic acid

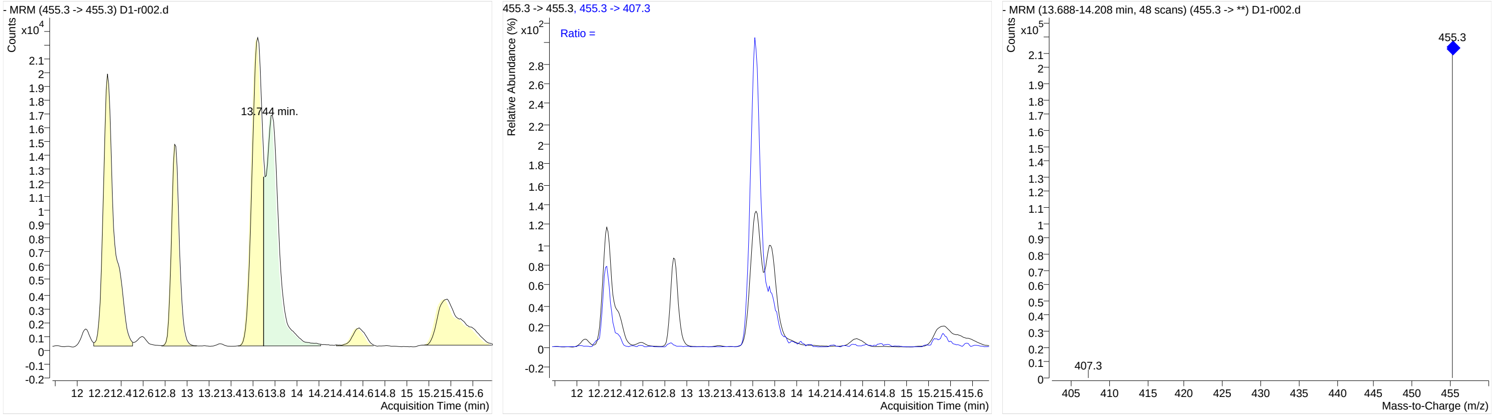

# Quantitative Analysis Complete Report

Agilent Technologies

| | | | |
|---|---|---|---|
| **Batch Path** | D:\MassHunter\Data\WBR\2022\20220127-PA25\QuantResults\1.batch.bin | | |
| **Analysis Time** | 1/29/2022 11:06:08 AM | **Analyst Name** | admin-PC\admin |
| **Report Time** | 1/29/2022 11:09:56 AM | **Reporter Name** | admin |
| **Last Calib Update** | 1/29/2022 11:06:07 AM | **Batch State** | Processed |
| **Quant Batch Version** | B.07.01SP1 | **Quant Report Version** | B.07.01 SP1 |

| | | | |
|---|---|---|---|
| **Acq. Time** | 1/28/2022 1:18:56 AM | **Data File** | D1-r003.d |
| **Sample Type** | Sample | **Sample Name** | D1 |
| **Dilution** | 1 | **Acq. Method** | 20220127-PA25.m |

## Sample Chromatogram

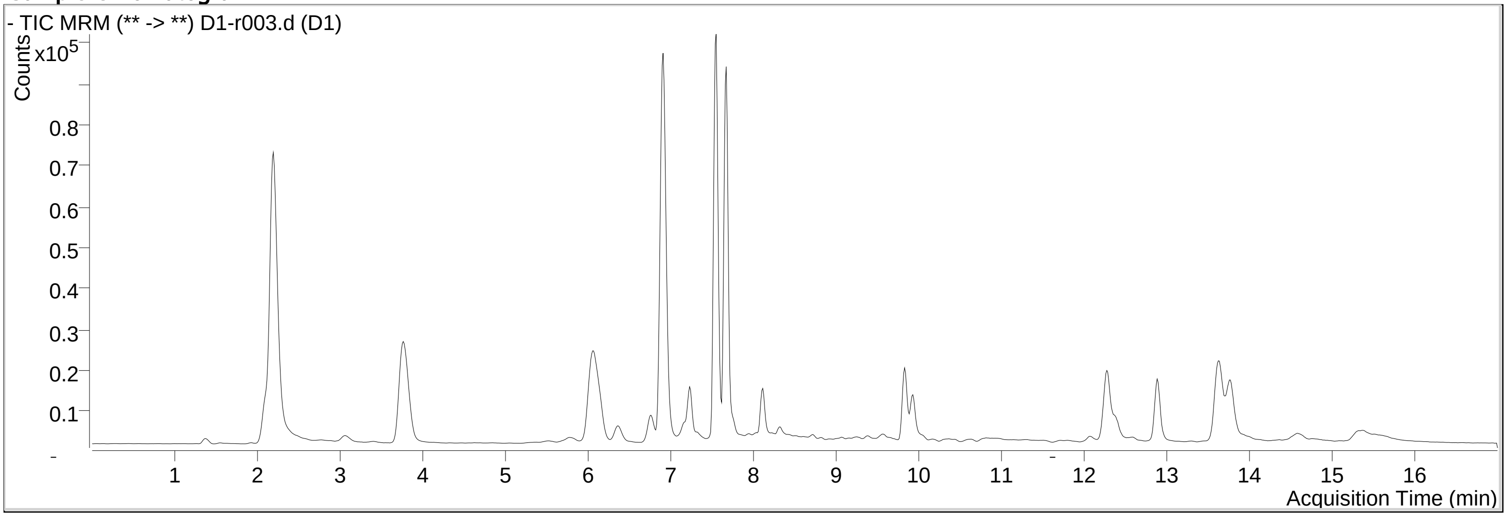


## 24-Ursolic acid

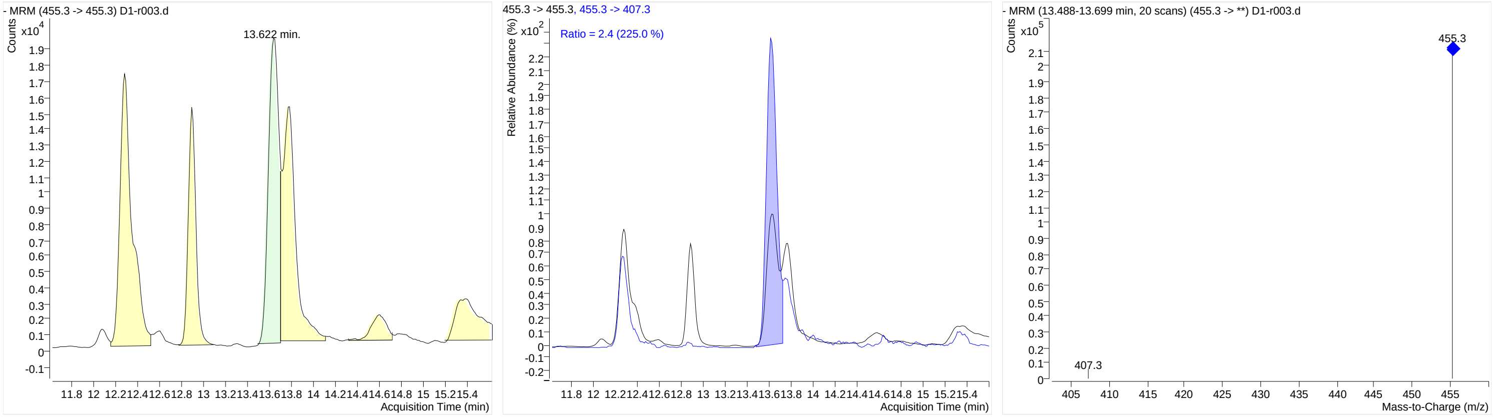


## 25-Oleanolic acid

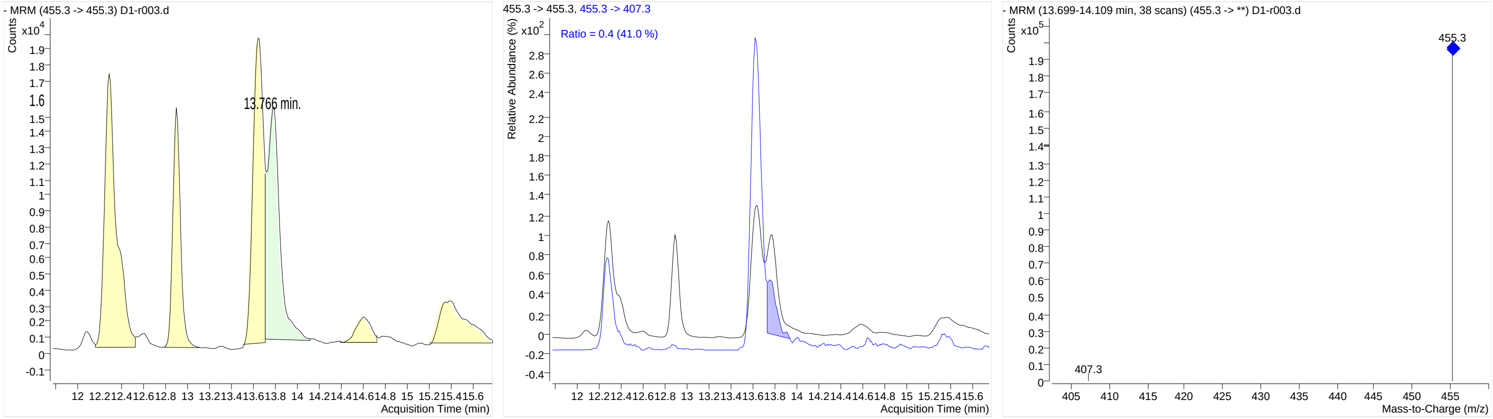

# Quantitative Analysis Complete Report

| | | | |
|---|---|---|---|
| **Batch Path** | D:\MassHunter\Data\WBR\2022\20220127-PA25\QuantResults\1.batch.bin | | |
| **Analysis Time** | 1/29/2022 11:06:08 AM | **Analyst Name** | admin-PC\admin |
| **Report Time** | 1/29/2022 11:09:59 AM | **Reporter Name** | admin |
| **Last Calib Update** | 1/29/2022 11:06:07 AM | **Batch State** | Processed |
| **Quant Batch Version** | B.07.01SP1 | **Quant Report Version** | B.07.01 SP1 |

| | | | |
|---|---|---|---|
| **Acq. Time** | 1/28/2022 1:36:39 AM | **Data File** | D2.d |
| **Sample Type** | Sample | **Sample Name** | D2 |
| **Dilution** | 1 | **Acq. Method** | 20220127-PA25.m |

## Sample Chromatogram

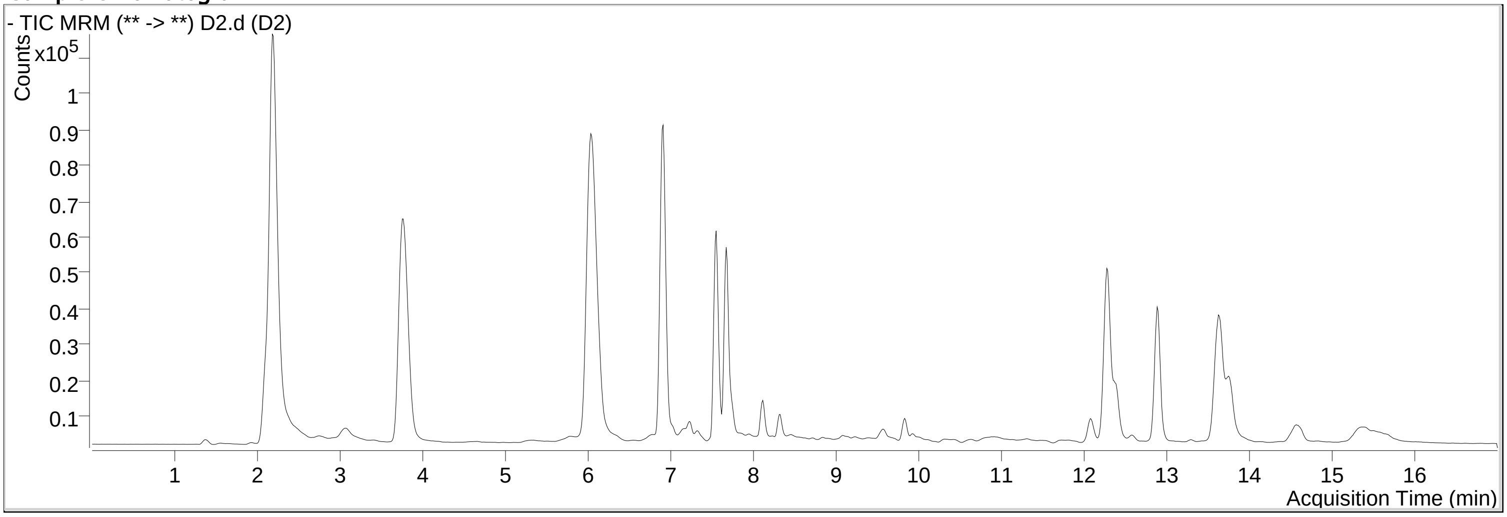


## 24-Ursolic acid

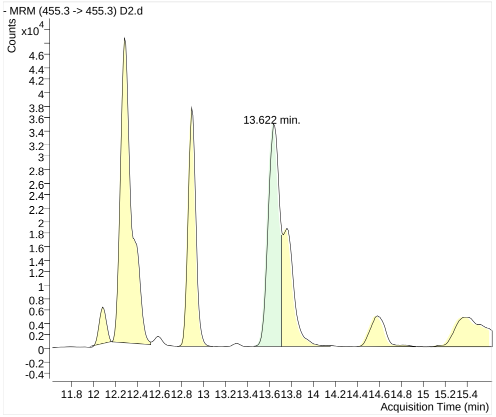


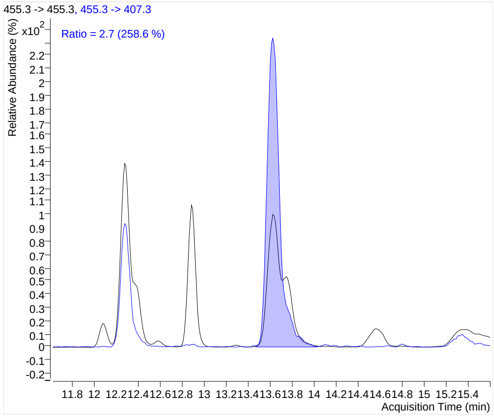


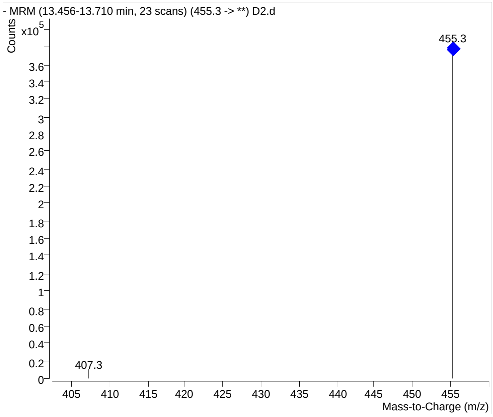


## 25-Oleanolic acid

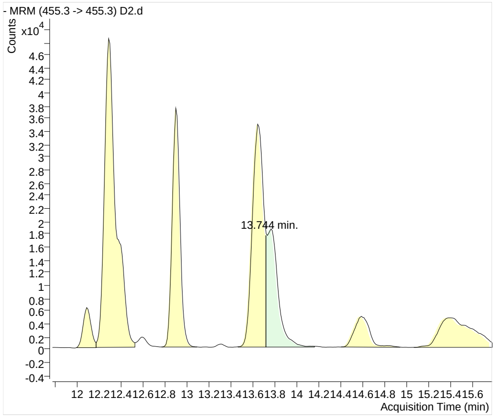


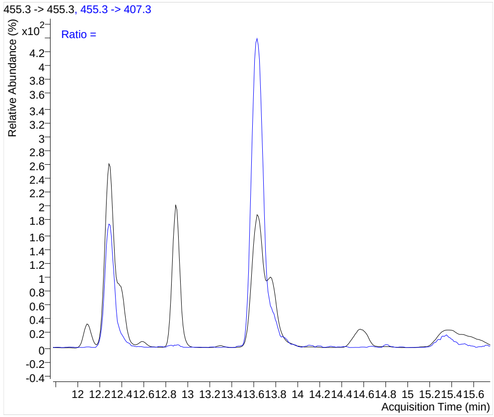


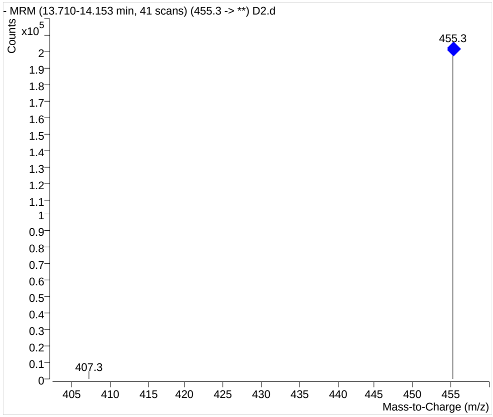

# Quantitative Analysis Complete Report

| | | | |
|---|---|---|---|
| **Batch Path** | D:\MassHunter\Data\WBR\2022\20220127-PA25\QuantResults\1.batch.bin | | |
| **Analysis Time** | 1/29/2022 11:06:08 AM | **Analyst Name** | admin-PC\admin |
| **Report Time** | 1/29/2022 11:10:02 AM | **Reporter Name** | admin |
| **Last Calib Update** | 1/29/2022 11:06:07 AM | **Batch State** | Processed |
| **Quant Batch Version** | B.07.01SP1 | **Quant Report Version** | B.07.01 SP1 |

| | | | |
|---|---|---|---|
| **Acq. Time** | 1/28/2022 1:54:23 AM | **Data File** | D3.d |
| **Sample Type** | Sample | **Sample Name** | D3 |
| **Dilution** | 1 | **Acq. Method** | 20220127-PA25.m |

## Sample Chromatogram

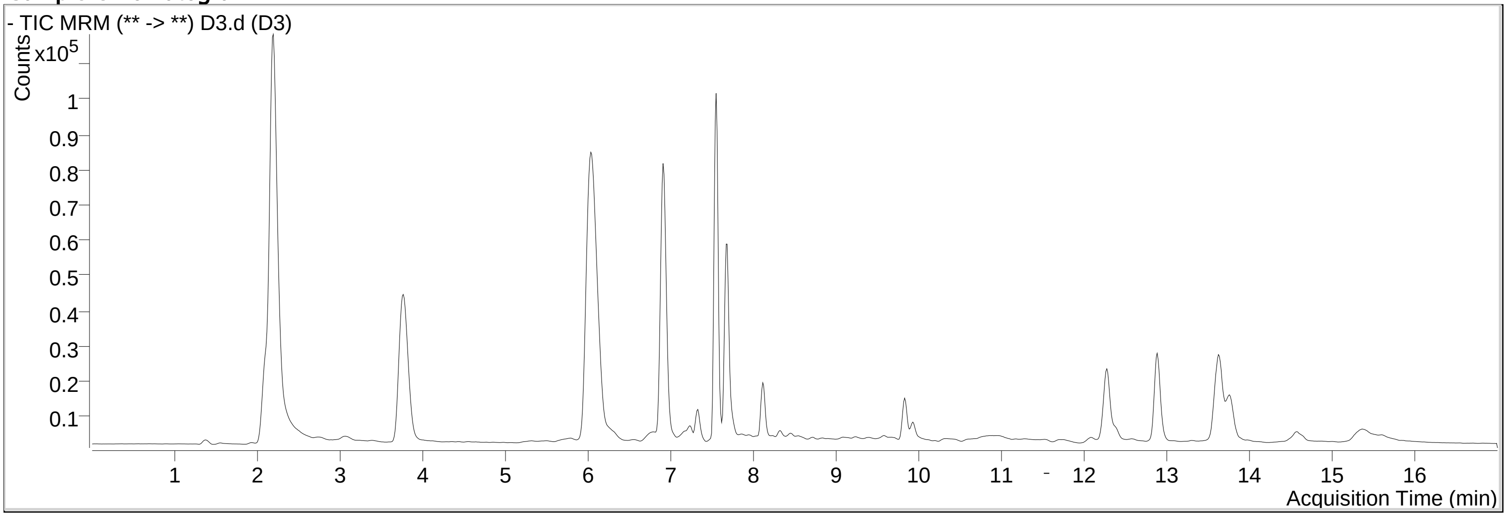


## 24-Ursolic acid

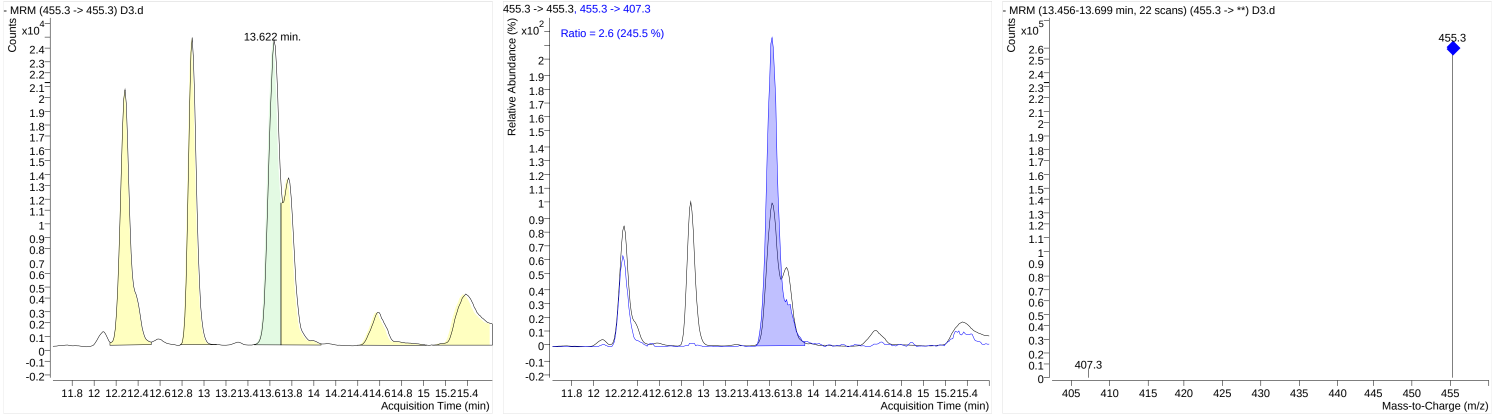


Acquisition Time (min) Acquisition Time (min) Mass-to-Charge (m/z)

## 25-Oleanolic acid

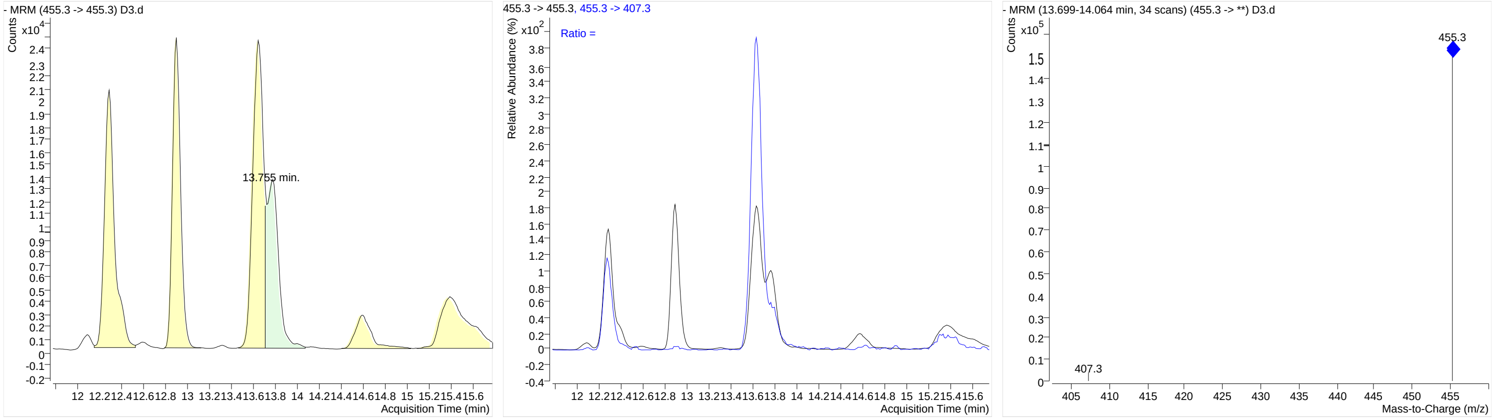

# Quantitative Analysis Complete Report

| | | | |
|---|---|---|---|
| **Batch Path** | D:\MassHunter\Data\WBR\2022\20220127-PA25\QuantResults\1.batch.bin | | |
| **Analysis Time** | 1/29/2022 11:06:08 AM | **Analyst Name** | admin-PC\admin |
| **Report Time** | 1/29/2022 11:10:06 AM | **Reporter Name** | admin |
| **Last Calib Update** | 1/29/2022 11:06:07 AM | **Batch State** | Processed |
| **Quant Batch Version** | B.07.01SP1 | **Quant Report Version** | B.07.01 SP1 |

| | | | |
|---|---|---|---|
| **Acq. Time** | 1/28/2022 2:12:05 AM | **Data File** | D4.d |
| **Sample Type** | Sample | **Sample Name** | D4 |
| **Dilution** | 1 | **Acq. Method** | 20220127-PA25.m |

## Sample Chromatogram

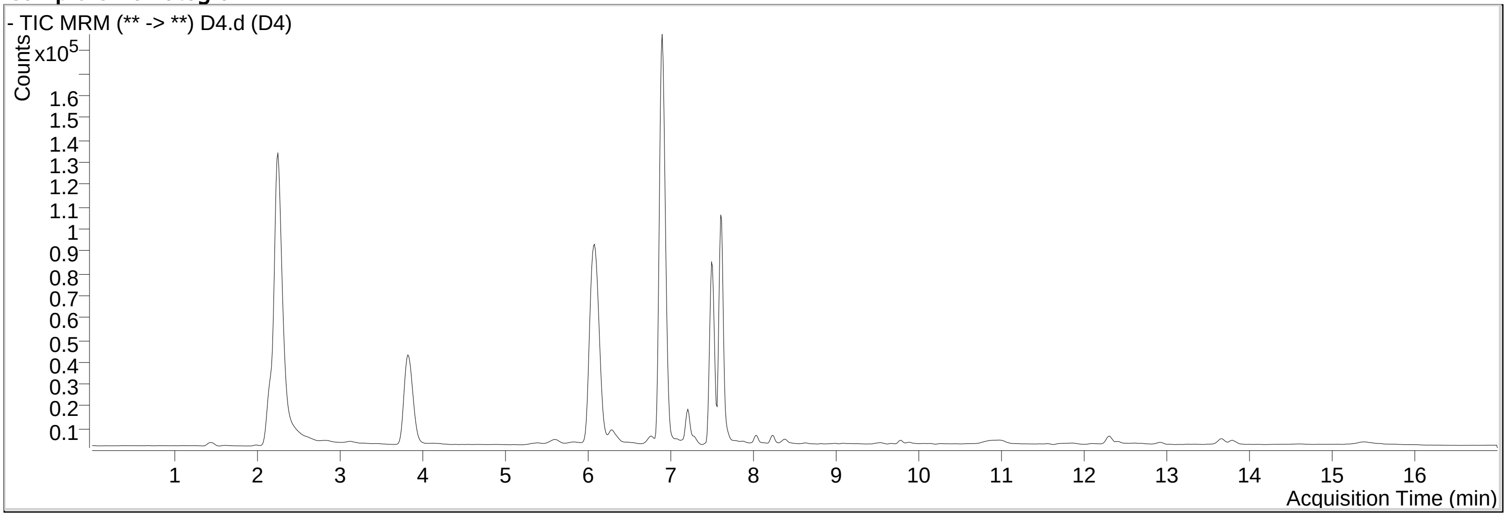


## 24-Ursolic acid

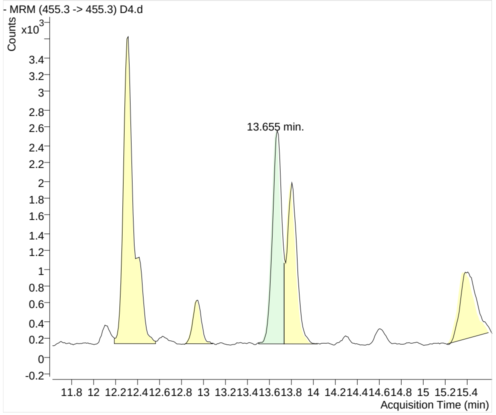


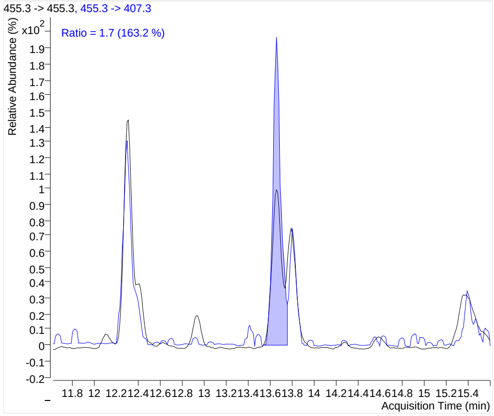


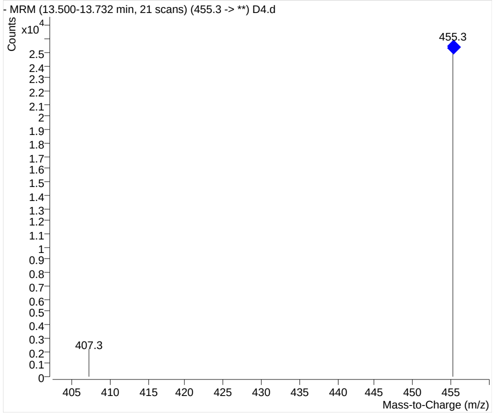


## 25-Oleanolic acid

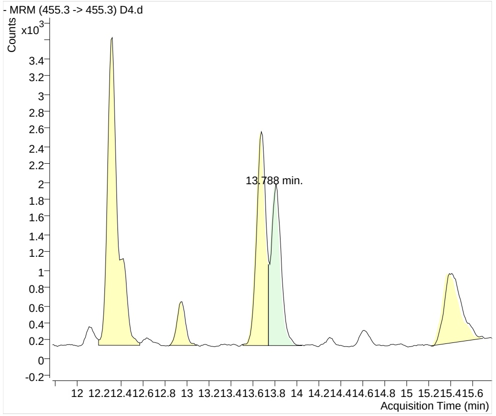


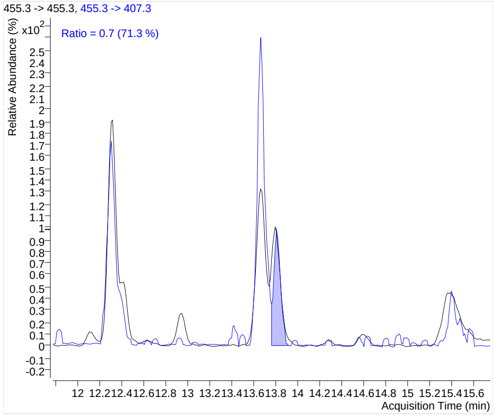


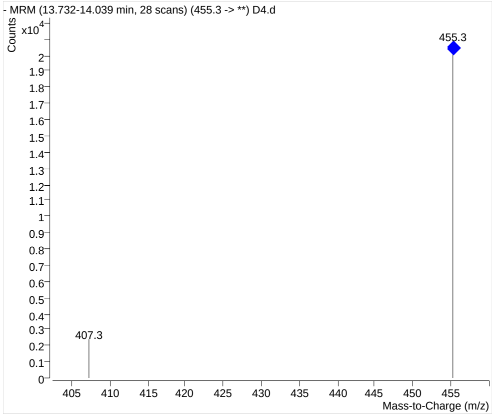

# Quantitative Analysis Complete Report

| | | | |
|---|---|---|---|
| **Batch Path** | D:\MassHunter\Data\WBR\2022\20220127-PA25\QuantResults\1.batch.bin | | |
| **Analysis Time** | 1/29/2022 11:06:08 AM | **Analyst Name** | admin-PC\admin |
| **Report Time** | 1/29/2022 11:10:09 AM | **Reporter Name** | admin |
| **Last Calib Update** | 1/29/2022 11:06:07 AM | **Batch State** | Processed |
| **Quant Batch Version** | B.07.01SP1 | **Quant Report Version** | B.07.01 SP1 |

| | | | |
|---|---|---|---|
| **Acq. Time** | 1/28/2022 2:29:50 AM | **Data File** | D5.d |
| **Sample Type** | Sample | **Sample Name** | D5 |
| **Dilution** | 1 | **Acq. Method** | 20220127-PA25.m |

## Sample Chromatogram

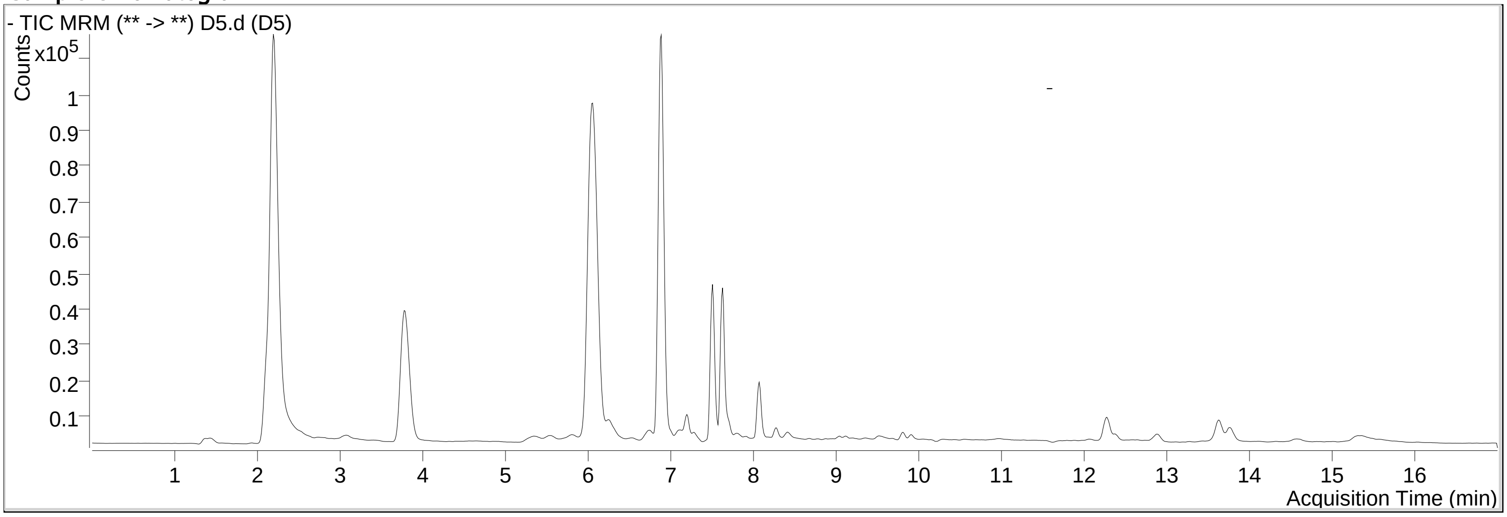


## 24-Ursolic acid

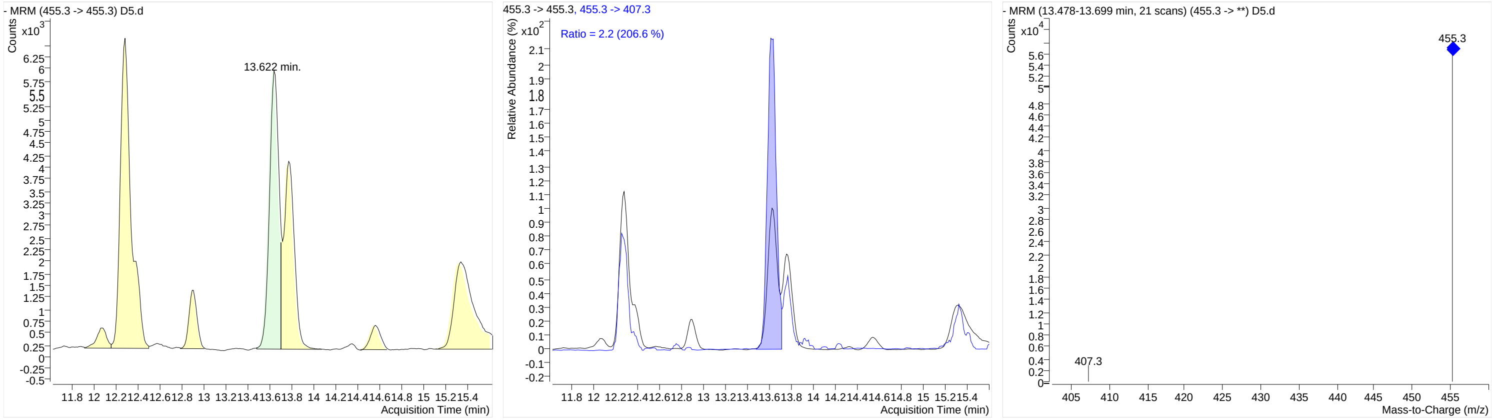


## 25-Oleanolic acid

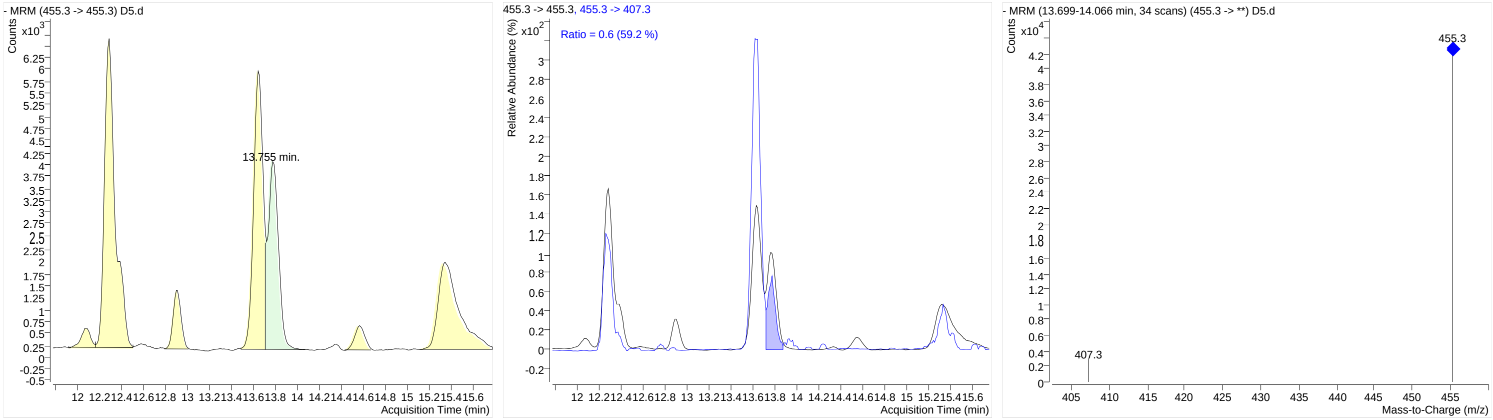

Agilent Technologies

| | | | |
|---|---|---|---|
| **Batch Path** | D:\MassHunter\Data\WBR\2022\20220127-PA25\QuantResults\1.batch.bin | | |
| **Analysis Time** | 1/29/2022 11:06:08 AM | **Analyst Name** | admin-PC\admin |
| **Report Time** | 1/29/2022 11:10:12 AM | **Reporter Name** | admin |
| **Last Calib Update** | 1/29/2022 11:06:07 AM | **Batch State** | Processed |
| **Quant Batch Version** | B.07.01SP1 | **Quant Report Version** | B.07.01 SP1 |

| | | | |
|---|---|---|---|
| **Acq. Time** | 1/28/2022 2:47:32 AM | **Data File** | D6.d |
| **Sample Type** | Sample | **Sample Name** | D6 |
| **Dilution** | 1 | **Acq. Method** | 20220127-PA25.m |

## Sample Chromatogram

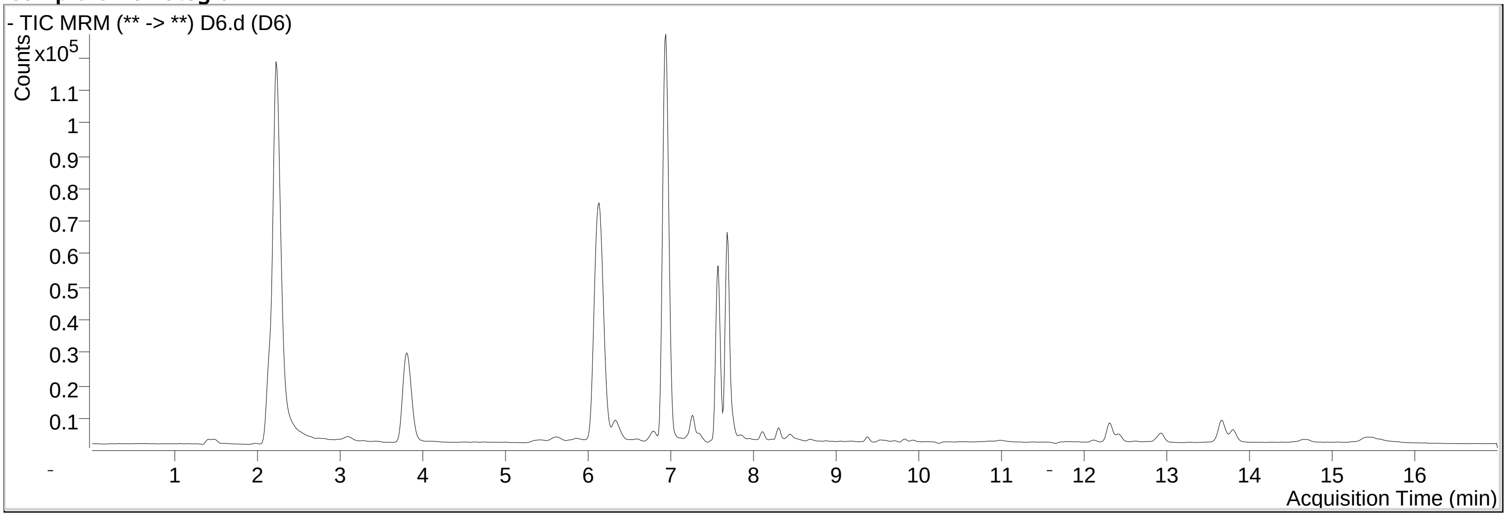


## 24-Ursolic acid

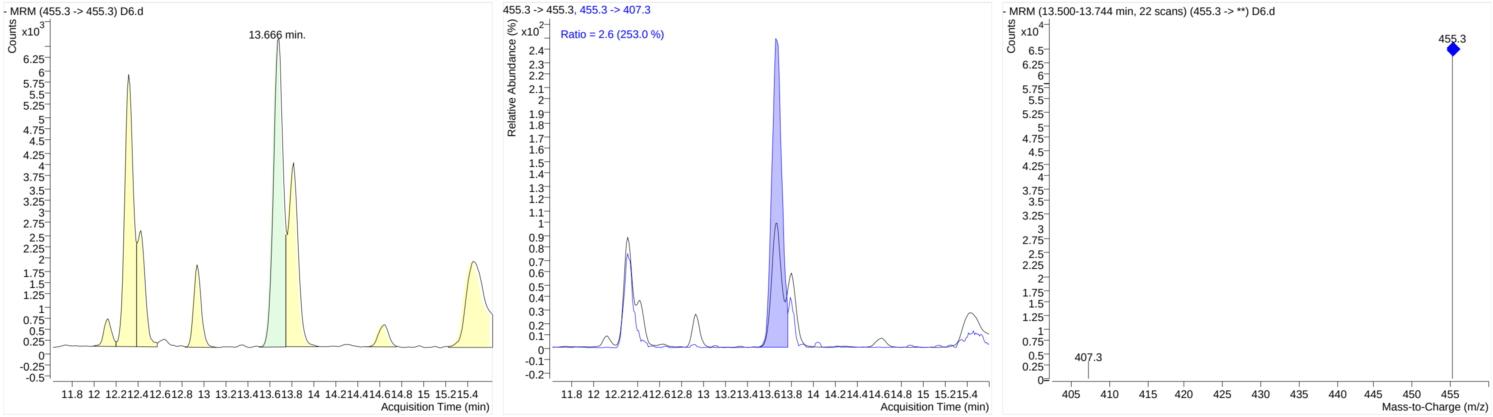


## 25-Oleanolic acid

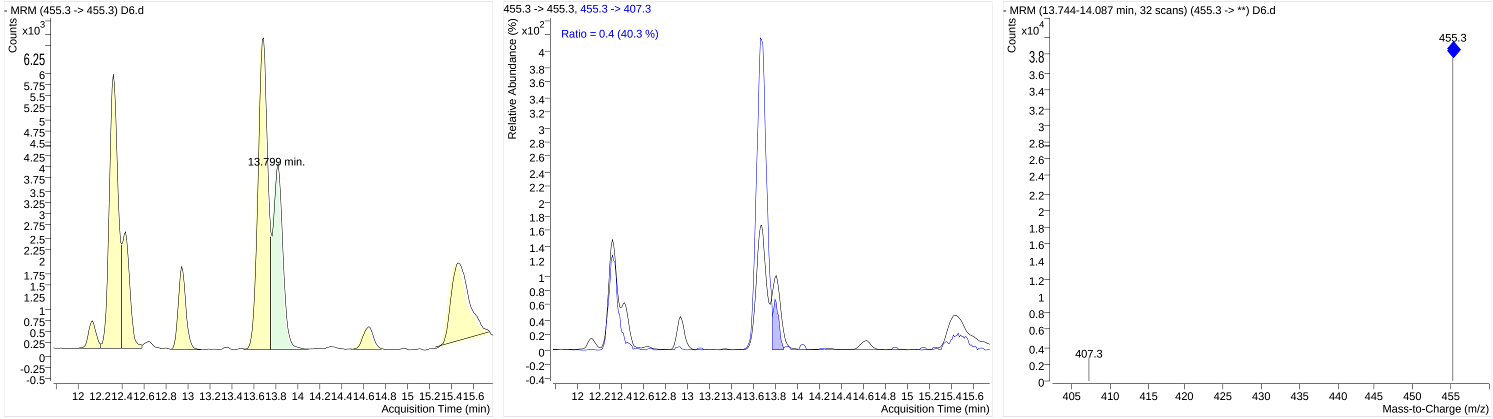

# Quantitative Analysis Complete Report

Agilent Technologies

| | | | |
|---|---|---|---|
| **Batch Path** | D:\MassHunter\Data\WBR\2022\20220127-PA25\QuantResults\1.batch.bin | | |
| **Analysis Time** | 1/29/2022 11:06:08 AM | **Analyst Name** | admin-PC\admin |
| **Report Time** | 1/29/2022 11:10:16 AM | **Reporter Name** | admin |
| **Last Calib Update** | 1/29/2022 11:06:07 AM | **Batch State** | Processed |
| **Quant Batch Version** | B.07.01SP1 | **Quant Report Version** | B.07.01 SP1 |

| | | | |
|---|---|---|---|
| **Acq. Time** | 1/28/2022 3:05:13 AM | **Data File** | D7.d |
| **Sample Type** | Sample | **Sample Name** | D7 |
| **Dilution** | 1 | **Acq. Method** | 20220127-PA25.m |

## Sample Chromatogram

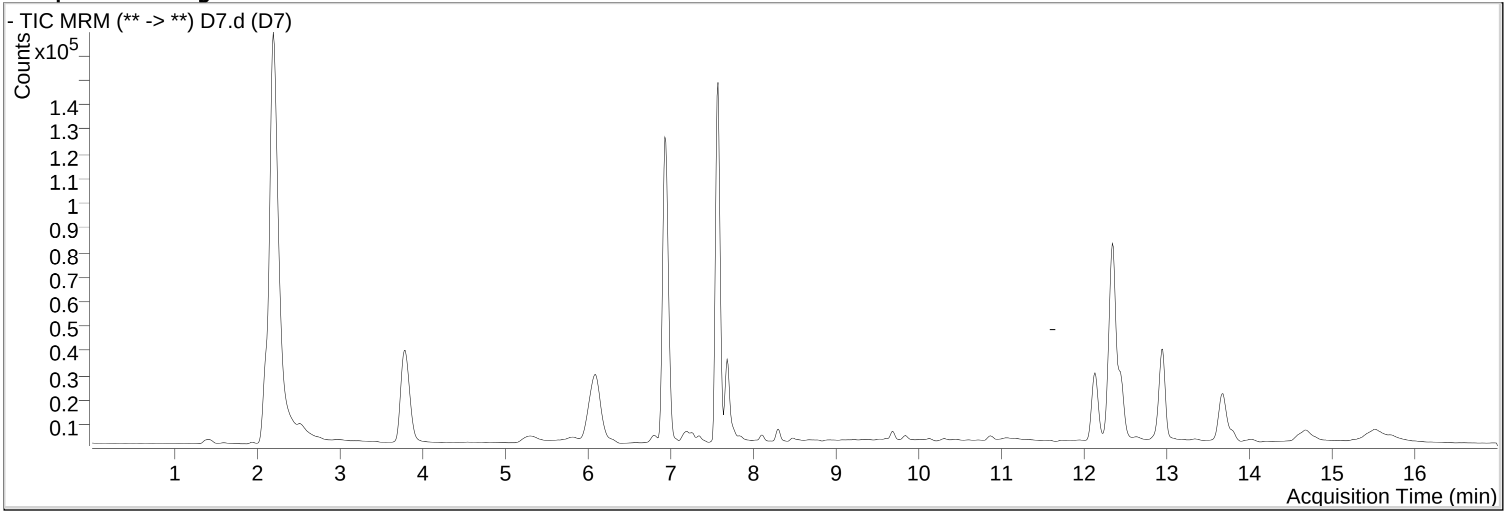


## 24-Ursolic acid

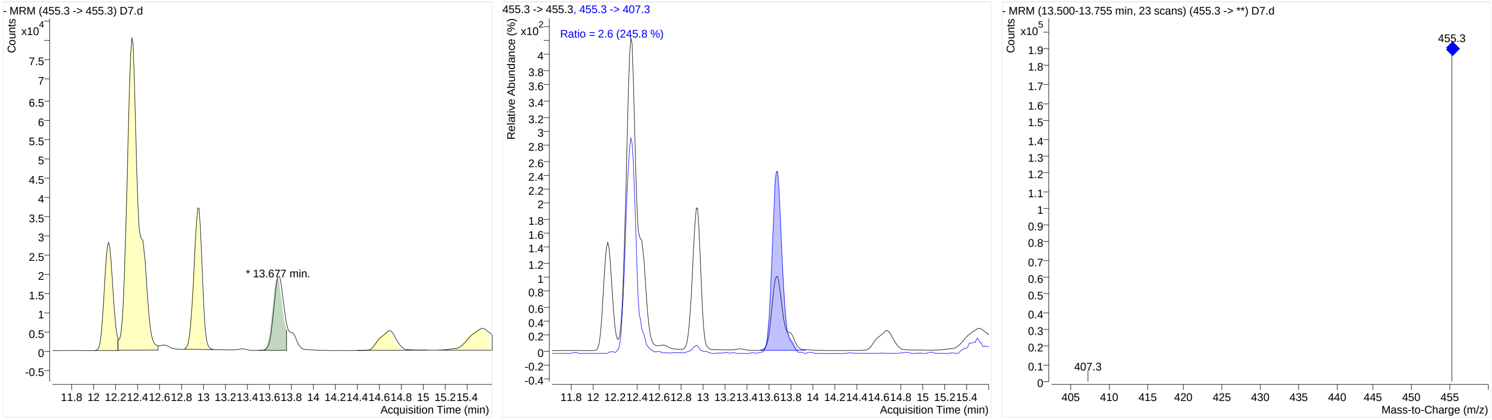


## 25-Oleanolic acid

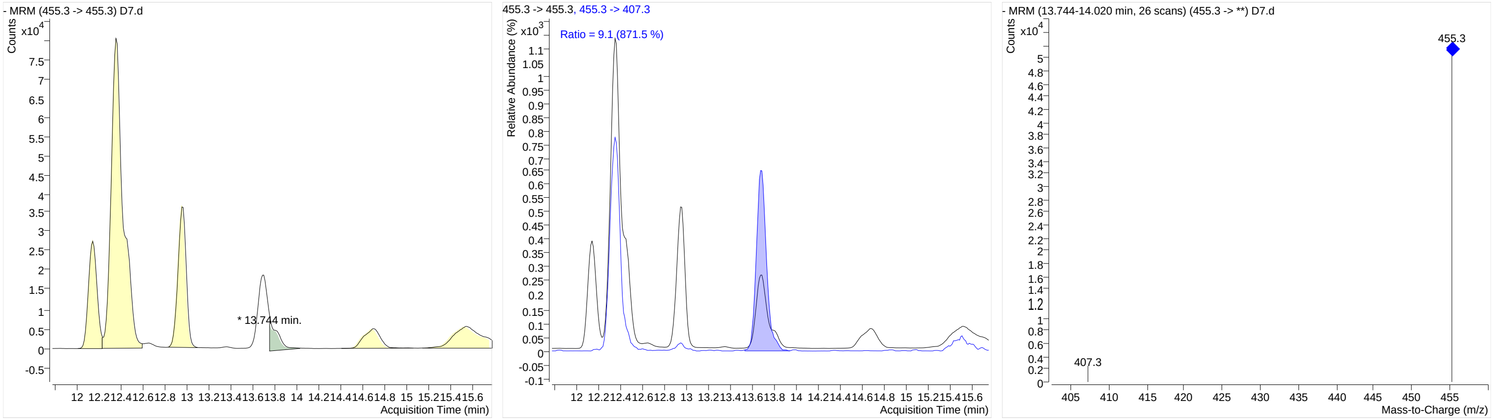

# Quantitative Analysis Complete Report

| | | | |
|---|---|---|---|
| **Batch Path** | D:\MassHunter\Data\WBR\2022\20220127-PA25\QuantResults\1.batch.bin | | |
| **Analysis Time** | 1/29/2022 11:06:08 AM | **Analyst Name** | admin-PC\admin |
| **Report Time** | 1/29/2022 11:10:19 AM | **Reporter Name** | admin |
| **Last Calib Update** | 1/29/2022 11:06:07 AM | **Batch State** | Processed |
| **Quant Batch Version** | B.07.01SP1 | **Quant Report Version** | B.07.01 SP1 |

| | | | |
|---|---|---|---|
| **Acq. Time** | 1/28/2022 3:22:55 AM | **Data File** | D8.d |
| **Sample Type** | Sample | **Sample Name** | D8 |
| **Dilution** | 1 | **Acq. Method** | 20220127-PA25.m |

## Sample Chromatogram

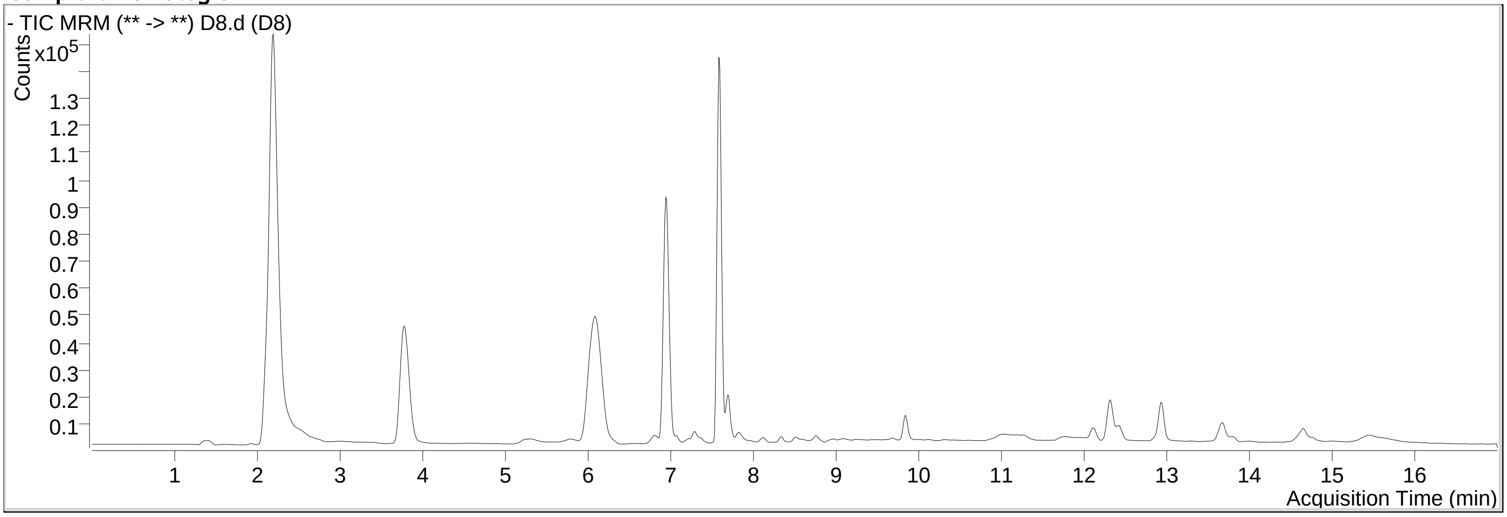


## 24-Ursolic acid

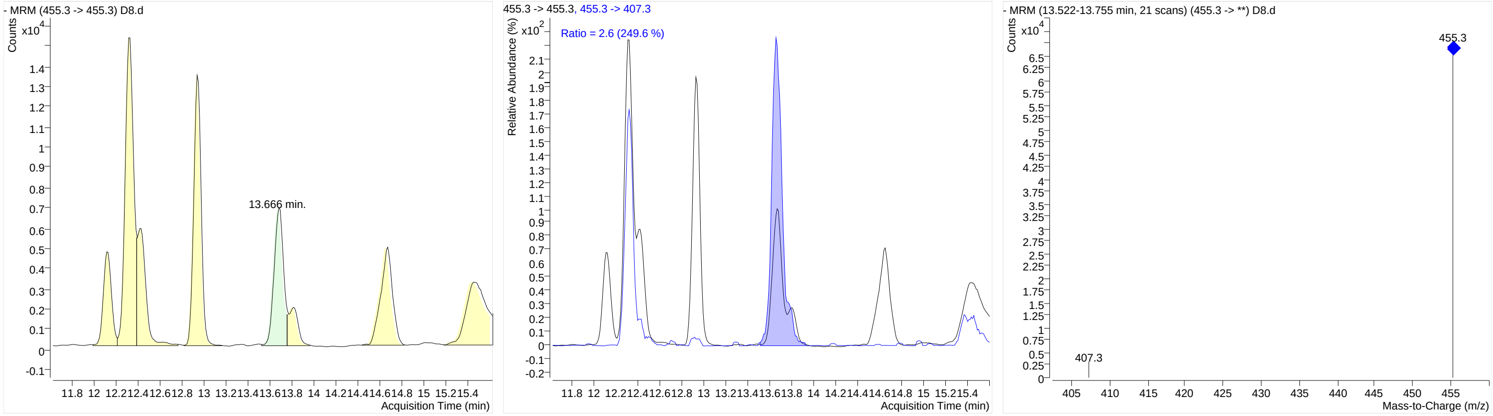


## 25-Oleanolic acid

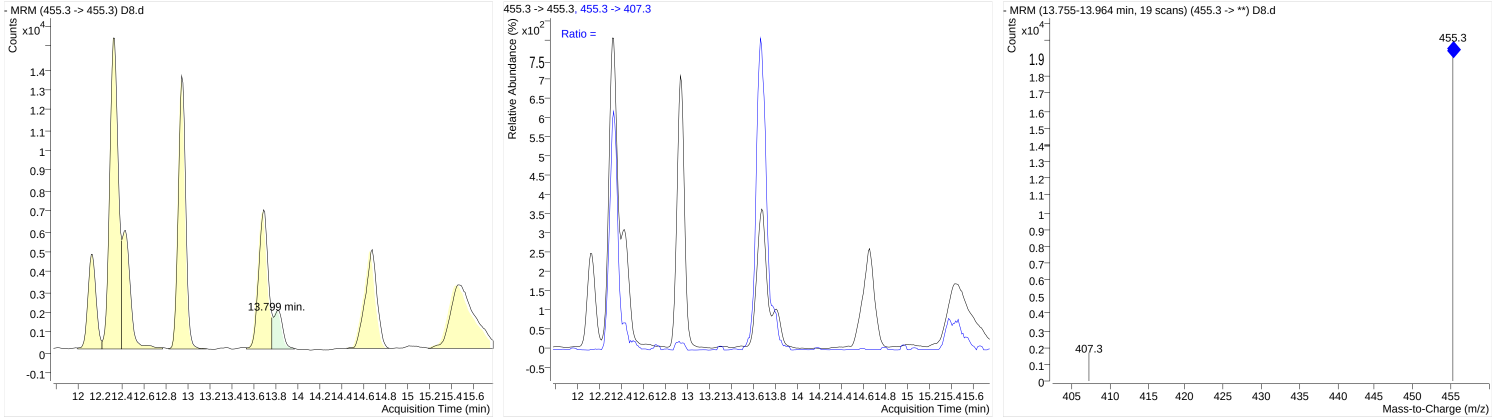

# Quantitative Analysis Complete Report

Agilent Technologies

| | | | |
|---|---|---|---|
| **Batch Path** | D:\MassHunter\Data\WBR\2022\20220127-PA25\QuantResults\1.batch.bin | | |
| **Analysis Time** | 1/29/2022 11:06:08 AM | **Analyst Name** | admin-PC\admin |
| **Report Time** | 1/29/2022 11:10:23 AM | **Reporter Name** | admin |
| **Last Calib Update** | 1/29/2022 11:06:07 AM | **Batch State** | Processed |
| **Quant Batch Version** | B.07.01SP1 | **Quant Report Version** | B.07.01 SP1 |

| | | | |
|---|---|---|---|
| **Acq. Time** | 1/28/2022 3:40:36 AM | **Data File** | D9.d |
| **Sample Type** | Sample | **Sample Name** | D9 |
| **Dilution** | 1 | **Acq. Method** | 20220127-PA25.m |

## Sample Chromatogram

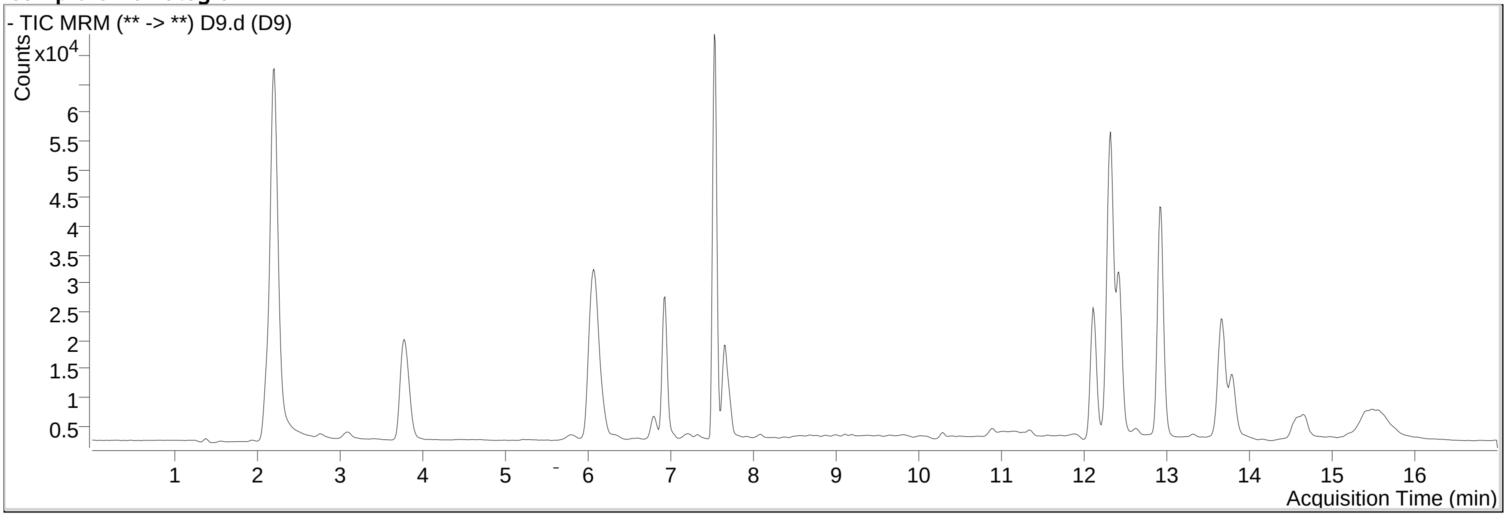


## 24-Ursolic acid

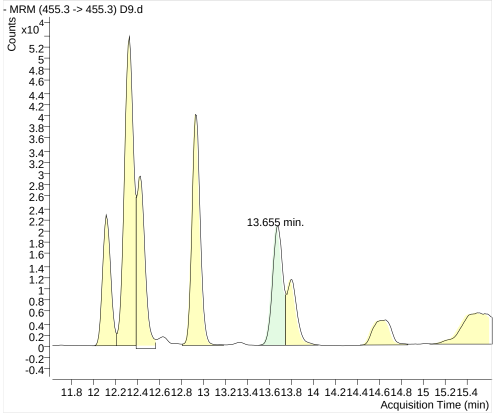


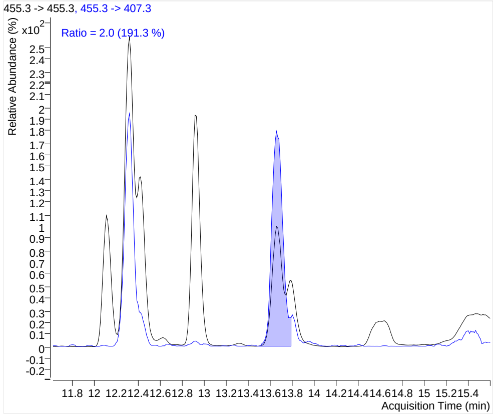


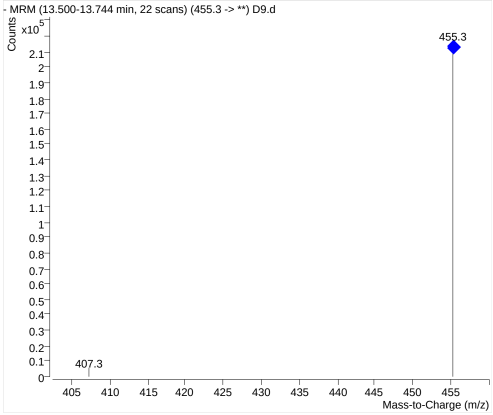


Acquisition Time (min) Acquisition Time (min) Mass-to-Charge (m/z)

## 25-Oleanolic acid

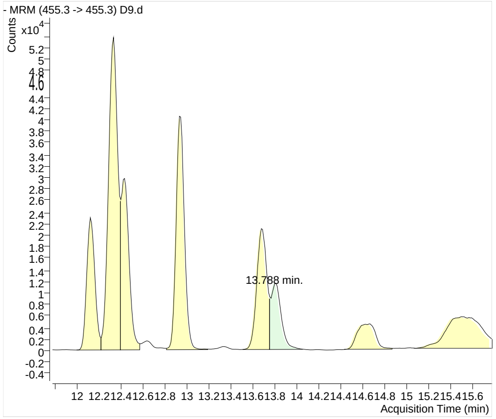


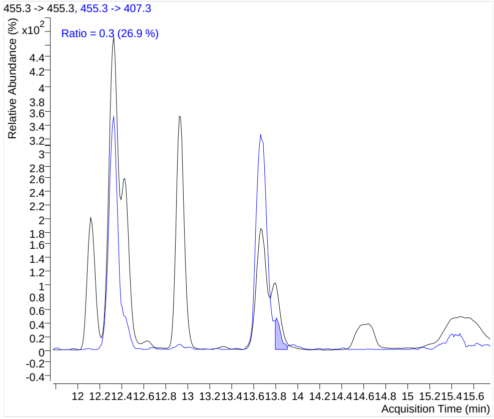


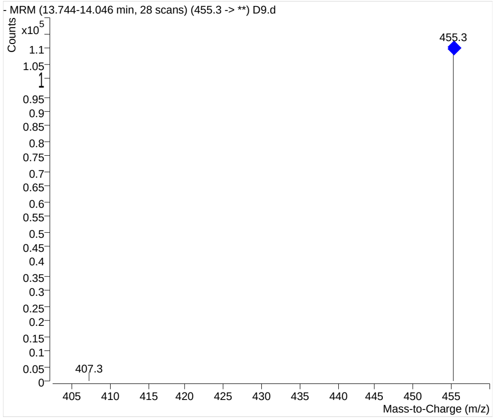

# Quantitative Analysis Complete Report

Agilent Technologies

| | | | |
|---|---|---|---|
| **Batch Path** | D:\MassHunter\Data\WBR\2022\20220127-PA25\QuantResults\1.batch.bin | | |
| **Analysis Time** | 1/29/2022 11:06:08 AM | **Analyst Name** | admin-PC\admin |
| **Report Time** | 1/29/2022 11:10:26 AM | **Reporter Name** | admin |
| **Last Calib Update** | 1/29/2022 11:06:07 AM | **Batch State** | Processed |
| **Quant Batch Version** | B.07.01SP1 | **Quant Report Version** | B.07.01 SP1 |

| | | | |
|---|---|---|---|
| **Acq. Time** | 1/28/2022 3:58:18 AM | **Data File** | D10.d |
| **Sample Type** | Sample | **Sample Name** | D10 |
| **Dilution** | 1 | **Acq. Method** | 20220127-PA25.m |

## Sample Chromatogram

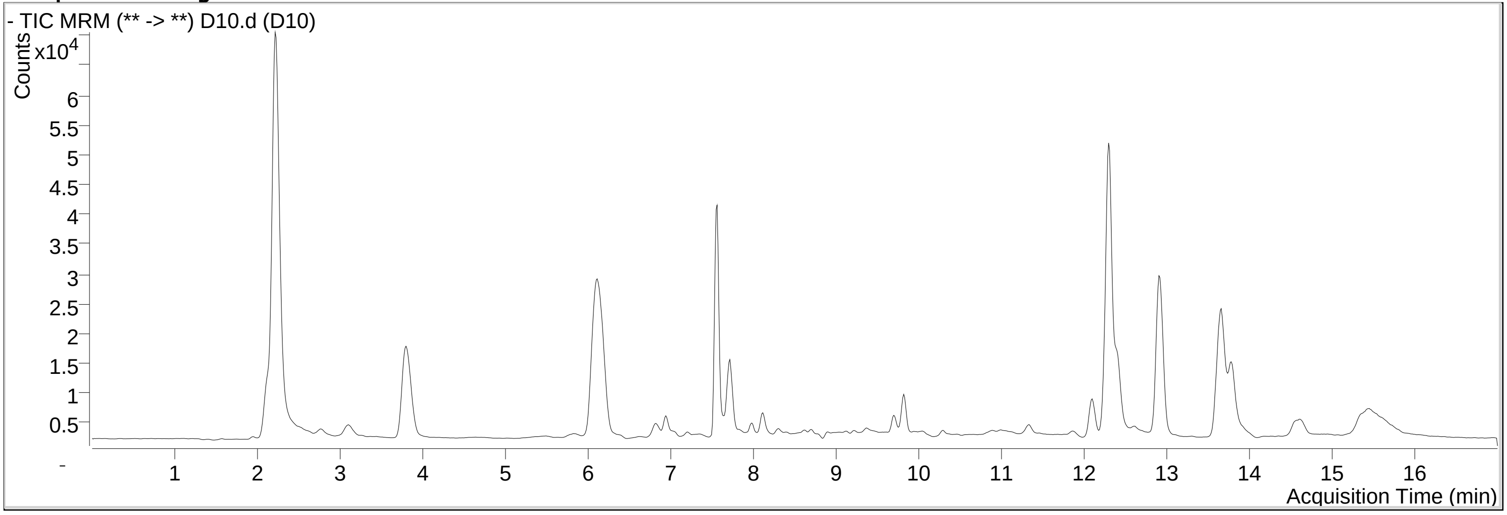


## 24-Ursolic acid

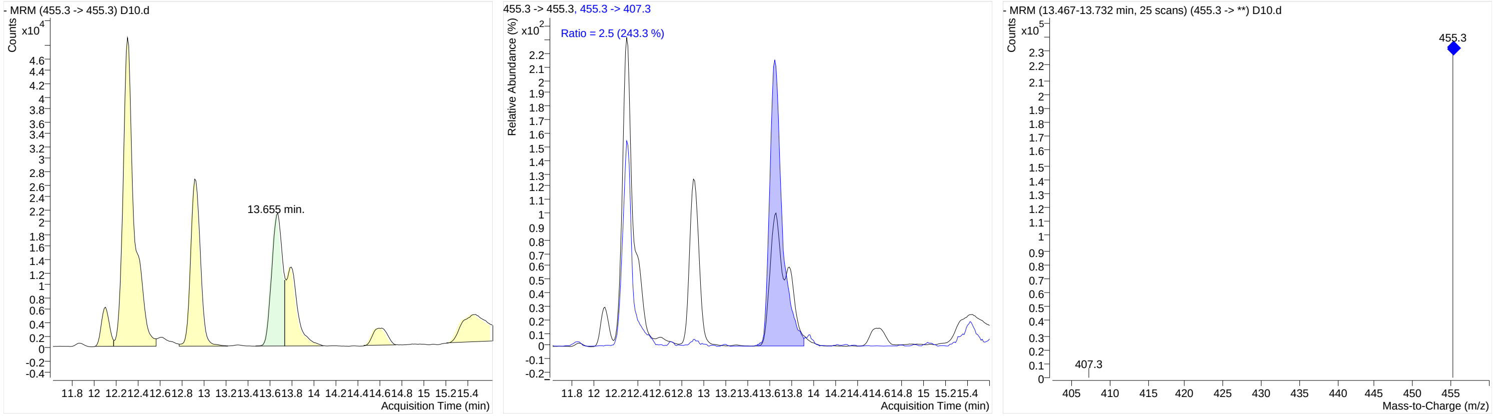


## 25-Oleanolic acid

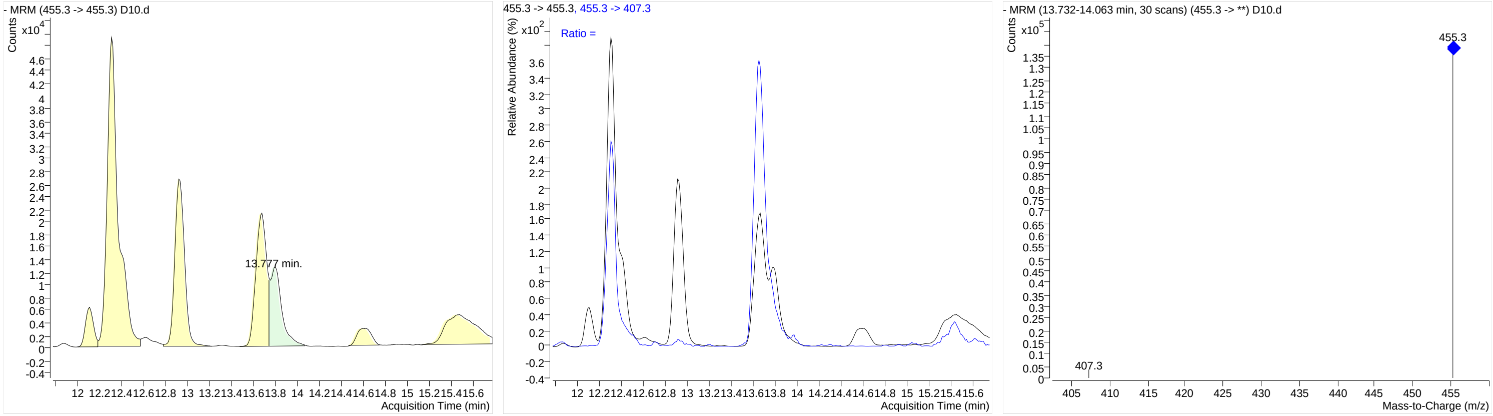

# Quantitative Analysis Complete Report

| | | | |
|---|---|---|---|
| **Batch Path** | D:\MassHunter\Data\WBR\2022\20220127-PA25\QuantResults\1.batch.bin | | |
| **Analysis Time** | 1/29/2022 11:06:08 AM | **Analyst Name** | admin-PC\admin |
| **Report Time** | 1/29/2022 11:10:29 AM | **Reporter Name** | admin |
| **Last Calib Update** | 1/29/2022 11:06:07 AM | **Batch State** | Processed |
| **Quant Batch Version** | B.07.01SP1 | **Quant Report Version** | B.07.01 SP1 |

| | | | |
|---|---|---|---|
| **Acq. Time** | 1/28/2022 4:15:57 AM | **Data File** | D11.d |
| **Sample Type** | Sample | **Sample Name** | D11 |
| **Dilution** | 1 | **Acq. Method** | 20220127-PA25.m |

## Sample Chromatogram

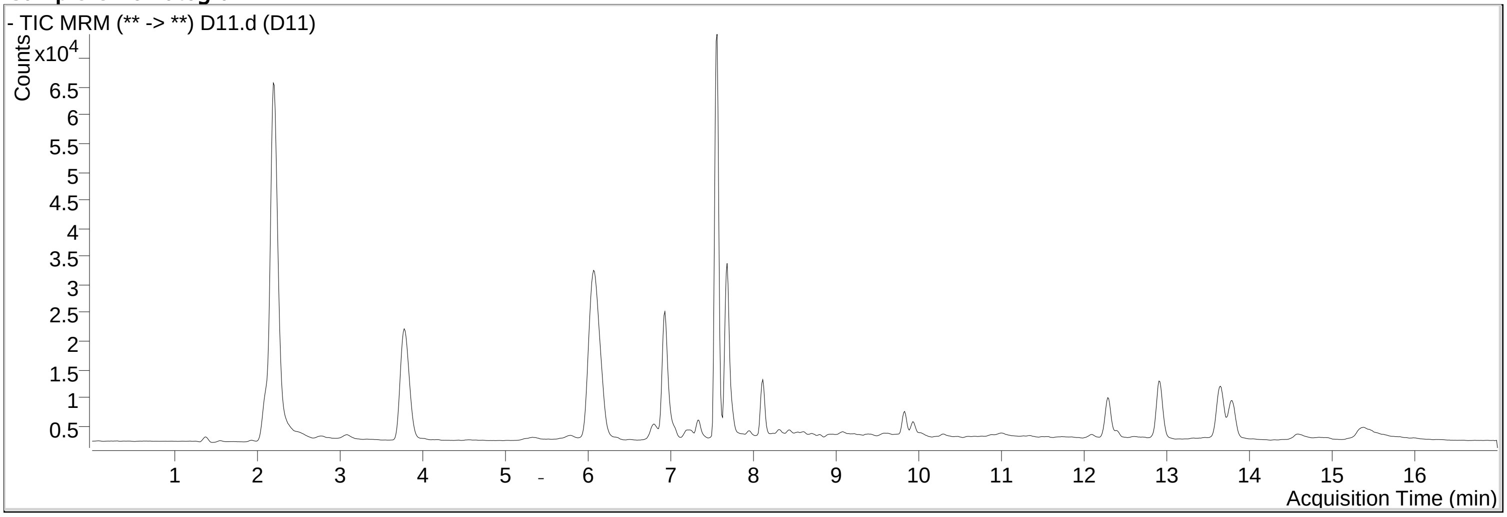


## 24-Ursolic acid

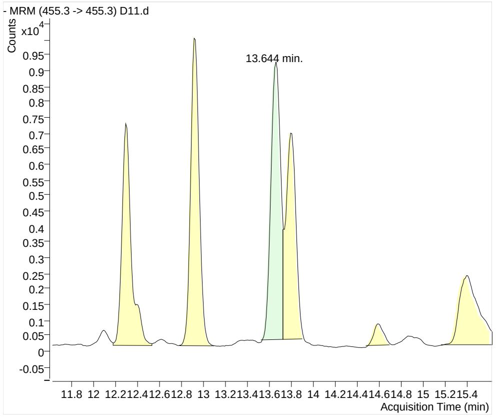


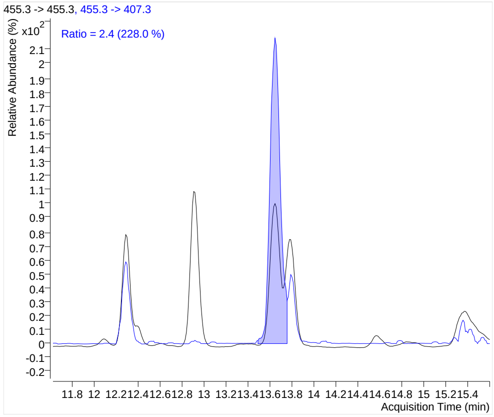


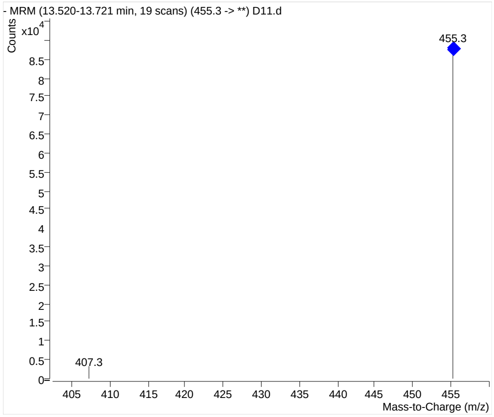


## 25-Oleanolic acid

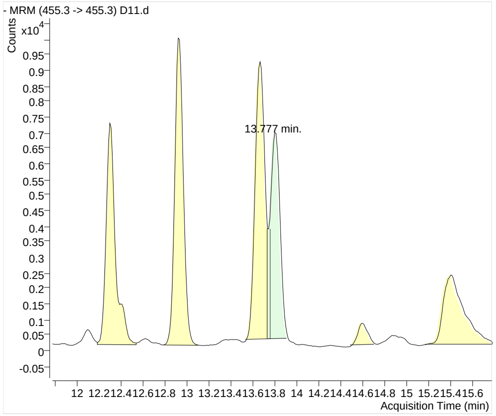


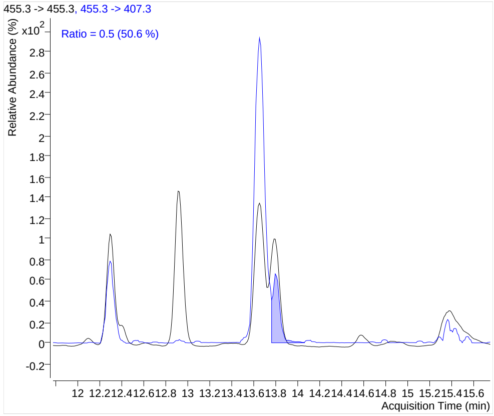


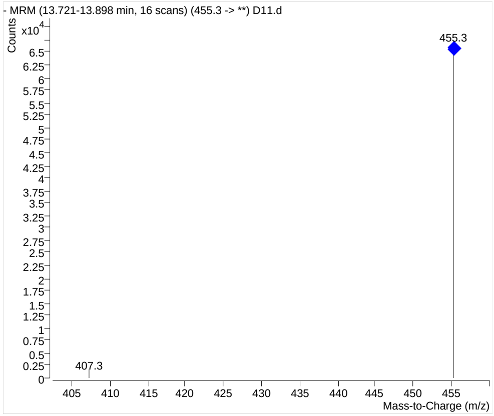

# Quantitative Analysis Complete Report

| | | | |
|---|---|---|---|
| **Batch Path** | D:\MassHunter\Data\WBR\2022\20220127-PA25\QuantResults\1.batch.bin | | |
| **Analysis Time** | 1/29/2022 11:06:08 AM | **Analyst Name** | admin-PC\admin |
| **Report Time** | 1/29/2022 11:10:33 AM | **Reporter Name** | admin |
| **Last Calib Update** | 1/29/2022 11:06:07 AM | **Batch State** | Processed |
| **Quant Batch Version** | B.07.01SP1 | **Quant Report Version** | B.07.01 SP1 |

| | | | |
|---|---|---|---|
| **Acq. Time** | 1/28/2022 4:33:38 AM | **Data File** | D12.d |
| **Sample Type** | Sample | **Sample Name** | D12 |
| **Dilution** | 1 | **Acq. Method** | 20220127-PA25.m |

**Sample Chromatogram**

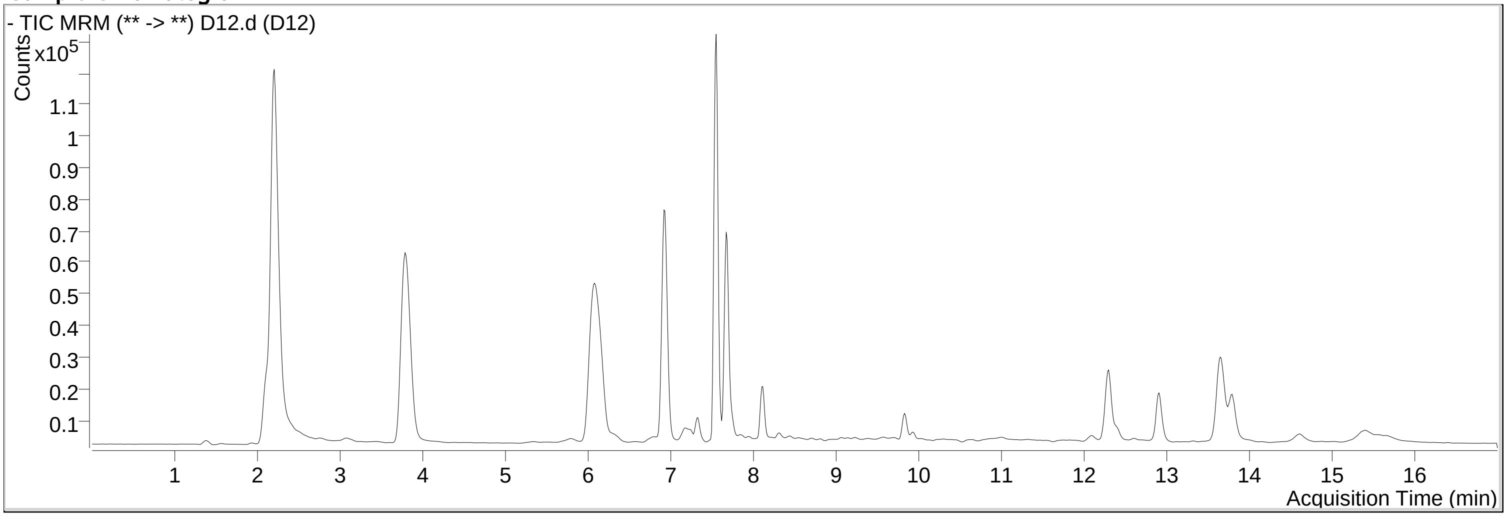


## 24-Ursolic acid

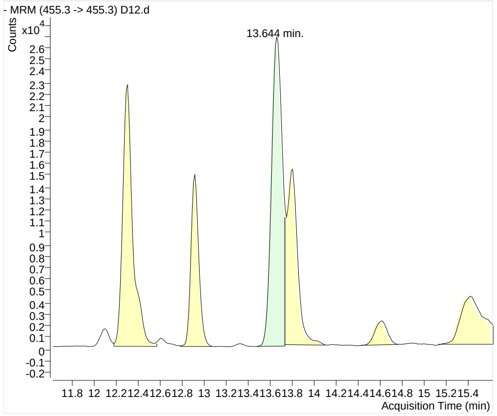


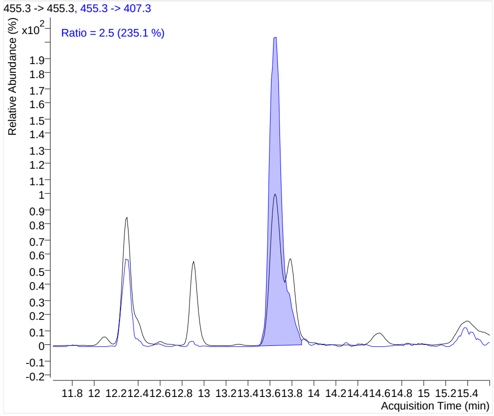


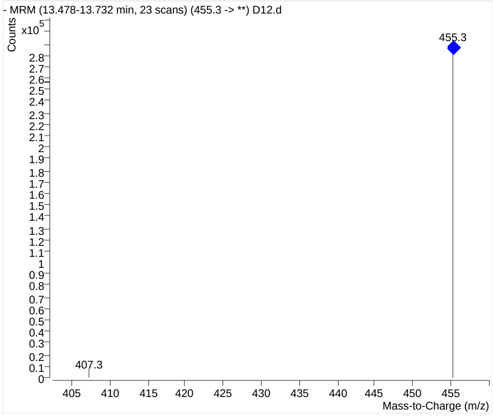


## 25-Oleanolic acid

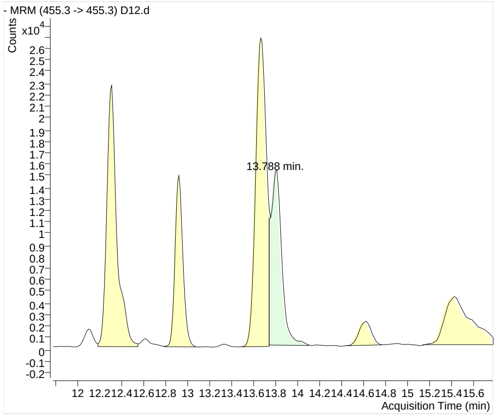


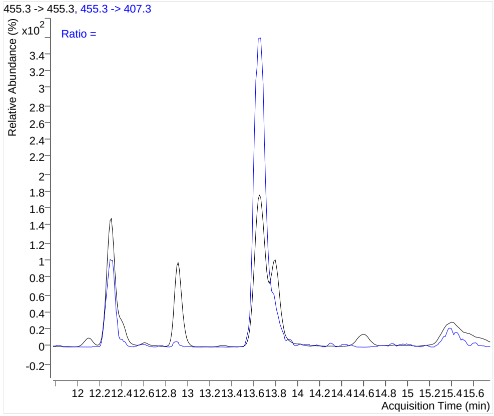


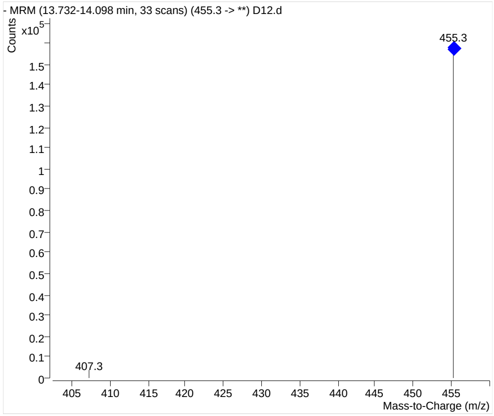


-

# Quantitative Analysis Complete Report

Agilent Technologies

| | | | |
|---|---|---|---|
| **Batch Path** | D:\MassHunter\Data\WBR\2022\20220127-PA25\QuantResults\1.batch.bin | | |
| **Analysis Time** | 1/29/2022 11:06:08 AM | **Analyst Name** | admin-PC\admin |
| **Report Time** | 1/29/2022 11:10:36 AM | **Reporter Name** | admin |
| **Last Calib Update** | 1/29/2022 11:06:07 AM | **Batch State** | Processed |
| **Quant Batch Version** | B.07.01SP1 | **Quant Report Version** | B.07.01 SP1 |

| | | | |
|---|---|---|---|
| **Acq. Time** | 1/28/2022 4:51:19 AM | **Data File** | D13.d |
| **Sample Type** | Sample | **Sample Name** | D13 |
| **Dilution** | 1 | **Acq. Method** | 20220127-PA25.m |

## Sample Chromatogram

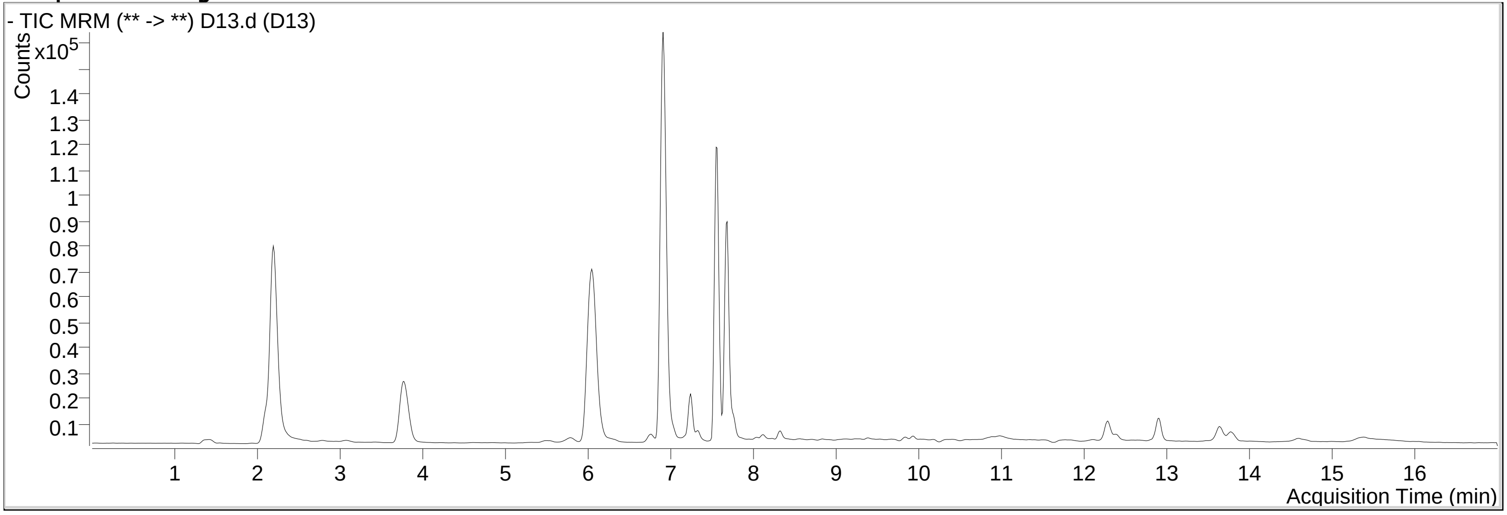


## 24-Ursolic acid

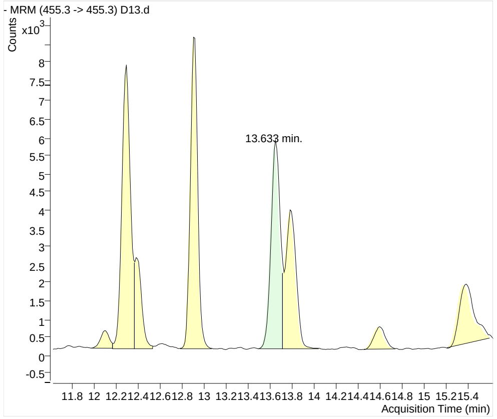


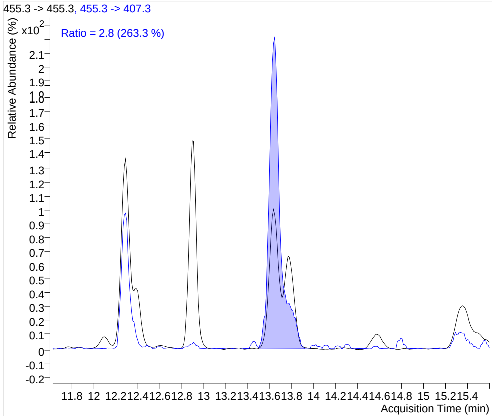


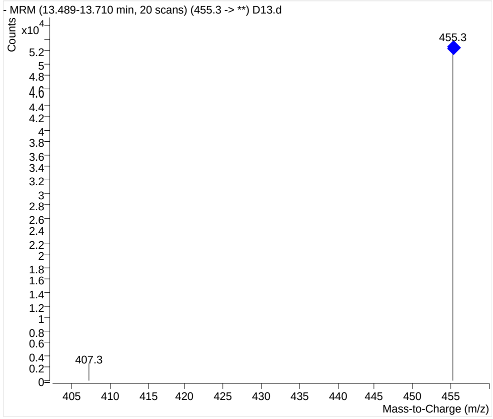


## 25-Oleanolic acid

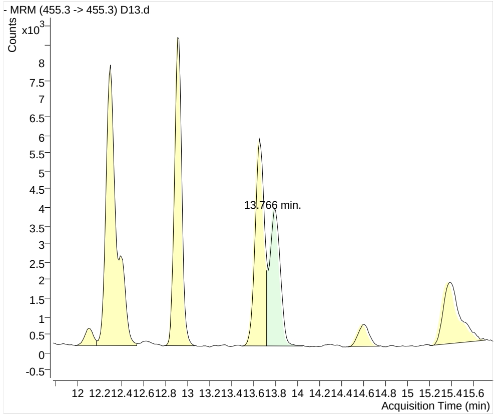


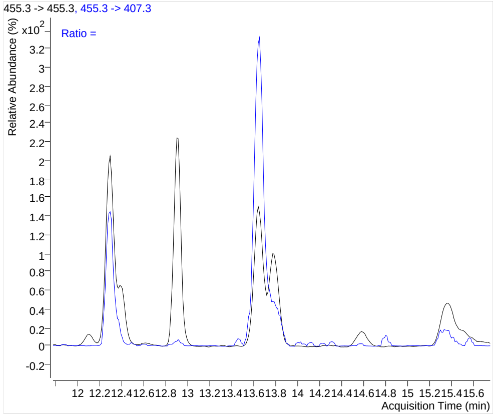


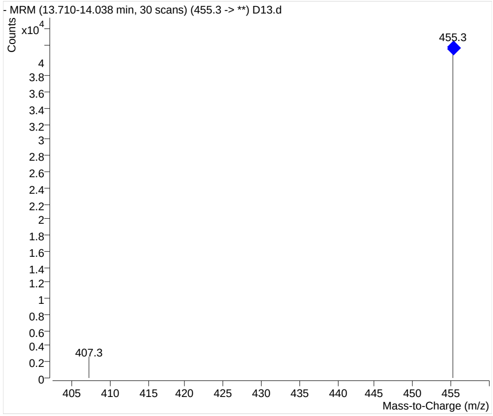

Supplement: Supplemental Information 3 [file peerj-11-15484-s003.pdf]
