# Supplementary material for: Influence of geographic origin and tissue type on the medicinal chemical compounds of Semiliquidambar cathayensis
Source: PeerJ. 2023 Jun 6;11:e15484. doi: 10.7717/peerj.15484 (PMC10252815; doi:10.7717/peerj.15484)
Supplement: Supplemental Information 4 [file peerj-11-15484-s004.docx]

| compounds | Mobile phase A | Mobile phase B | Wavelength of detection(nm) | Retention time(min) |
| --- | --- | --- | --- | --- |
| saikosaponin D | acetonitrile | 0.2% phosphate acid | 208 | 12.260 |
| catechin | acetonitrile | 0.17% acetic acid | 278 | 13.413 |
| paeoniflorin | acetonitrile | 0.1% phosphate acid | 274 | 14.350 |
| hesperidin | acetonitrile | 0.1% phosphate acid | 274 | 20.110 |
| kaempferol | methanol | 0.2% phosphate acid | 360 | 11.243 |
| isorhamnetin | methanol | 0.2% phosphate acid | 360 | 12.187 |
| keampferol-3-O-rutinoside | methanol | 0.5% phosphate acid | 360 | 3.693 |

**Supplementary data 2(MS analysis parameters of oleanolic acid and ursolic acid)**

| Compound | Parent ion(m/z) | Daughter ion(m/z) | Ionization mode | Collision energy(eV) |
| --- | --- | --- | --- | --- |
| oleanolic acid | 455.3 | 455.3*/407.3 | ESI- | 46 |
| ursolic acid | 455.3 | 455.3* | ESI- | 46 |

Note: * is quantitative ion
